# Gene-environment correlations and genetic confounding underlying the association between media use and mental health

**DOI:** 10.1038/s41598-022-25374-0

**Published:** 2023-01-19

**Authors:** Ziada Ayorech, Jessie R. Baldwin, Jean-Baptiste Pingault, Kaili Rimfeld, Robert Plomin

**Affiliations:** 1 Social, Genetic and Developmental Psychiatry Centre, Institute of Psychiatry, Psychology and Neuroscience, King’s College London, London, SE5 8AF UK; 2 Department of Psychology, PROMENTA Research Center, University of Oslo, Oslo, Norway; 3 Department of Clinical, Educational and Health Psychology, University College London, London, WC1H 0AP UK; 4 Department of Psychology, Royal Holloway University of London, London, TW20 0EX UK

**Keywords:** Behavioural genetics, Development

## Abstract

The increase in online media use and mental health problems have prompted investigations into their association, although most literature is focussed on deleterious effects. We assessed the aetiology of media use and mental health associations (*M* age = 22.14, SD = 0.85) using twin (n = 4000 pairs) and polygenic score methods (n = 6000 unrelated individuals) in the Twins Early Development Study. Beyond the traditionally explored negative uses of online media (online victimisation and problematic internet use), we investigate general media uses such as posting online and watching videos and distinguish both positive (pro-social behaviour) and negative (anxiety, depression, peer and behaviour problems) mental health measures. Negative media use correlated with poor mental health (r = 0.11–0.32), but general media use correlated with prosocial behaviour (*r* = 0.20) and fewer behavioural problems (r =  − 0.24). Twin analyses showed that both general and negative media use were moderately heritable (ranging from 20 to 49%) and their associations with mental health were primarily due to genetic influences (44–88%). Genetic sensitivity analysis combining polygenic scores with heritability estimates also suggest genetic confounding. Results indicate research on the mental health impact of media use should adopt genetically informed designs to strengthen causal inference.

## Introduction

Previous research on the negative impact of screen time focussed on time spent watching television. The dominant interpretation was that time spent watching television was not only negatively impacting obesity, sleep and skills^1–3^, but was also leading to poor educational achievement^4^, increased aggression^5,6^ and pervasive attention problems^7,8^. Correlations between television viewing and negative outcomes were often viewed as reflecting the causal impact of television on developmental outcomes^9^.

In 1990, the first genetically informed analysis of individual differences in time spent watching television challenged the notion that television is a pure ‘environmental’ factor^10^. Both parent–offspring and sibling adoption designs yielded evidence for substantial genetic influence on individual differences in television viewing, suggesting that young people are not passive recipients of their television environment but actively select, modify and interpret their experiences in line with their genetics^11^. The authors concluded that if television viewing shows substantial genetic influence, then it is possible that associations between behavioural outcomes and television viewing are in part, explained by genetic variation.

Decades later, the anxiety surrounding time spent on television has been replaced by concerns about time spent online^12^. Considerable research effort has been dedicated to understanding problematic mobile phone use and the extent to which excessive use and impulse control problems are sufficient to warrant a label of ‘addiction’^12,13^. It remains less understood to what extent mobile phone use shares the behavioural and neurobiological components typically associated with more established addictions. In addition, terminologies used to describe excessive phone use vary and include, but are not limited to ‘mobile phone addiction’, ‘problematic use’, ‘excessive use’, and ‘overuse of mobile phones’, and although ‘addictive’ like behavior associated with mobile phone use is a recognized problem, it is not recognized as a category in diagnostic manuals. Recent calls for better integration of the uniqueness of mobile phone use and the heterogeneity in its manifestation can be addressed by integrating individual differences into the framework, which is the goal of the present study.

Media use is one of the most influential environments during emerging adulthood^14,15^, a developmental stage in the late teens and early twenties marked by greater autonomy and identity exploration^14^. Online media use has become ubiquitous in developed countries with young adults spending more time on mobile phones than any other age group^16^. Most of the time spent on mobile phones does not involve making phone calls. Mobile phones serve many other functions such as sending messages, watching and recording videos, posting and sharing media content^17^. Crucially, online media serves as a constant source of connection to social media platforms, which are changing the landscape of social interactions and prompting new research avenues exploring the impact of online social connections on offline relations.

In contrast to television viewing at home, emerging adults have a great deal more autonomy about when they engage online, what they do online and how long they do it for. From a behavioural genetic perspective, this increased autonomy could be reflected in decreased estimates of shared environmental influence and increased estimates of genetic influence, as young people are able to tailor their media use to their individual interests, which we know to be genetically influenced^18^. We previously demonstrated that inherited DNA differences account for up to 40% of individual differences in time spent on educational media, entertaining media, gaming and the social network Facebook^19^. These results were based on a sample of over 2000 16-year-old twin pairs participating in the Twins Early Development Study (TEDS), a longitudinal birth cohort study of twin pairs born in England and Wales^20^. Online media use in these previous analyses was limited to home computer use before the first 4G network became available in the UK, which led to an exponential increase in use of mobile phones and other portable devices^21^. An updated exploration of genetic influence on mobile phone use is needed to reflect the profound changes in the digital environment of emerging adults.

The increase in young people’s online media use coincides with a substantial increase in self-reported mental health problems including greater demand for counselling services, more admissions for self-harm and more frequent referrals to mental health services^22–24^. Despite these reported increases, little is known about the role of social media use in these negative life outcomes^25,26^. Better understanding of the aetiology of online media use during early adulthood is needed given this life period has been acknowledged as a particular sensitive period for the emergence of mental health problems and a prodromal stage for which mental health interventions may be more effective. In addition, there has been an increased demand for more nuanced investigation of associations between media use and mental health, moving past a focus solely on the deleterious effects of online media use, to a more balanced view incorporating potential positive social media influences and individual differences^27^. It is timely to systematically investigate the relationship between diverse measures of online media use and mental health in emerging adulthood.

Genetically informed designs are needed to quantify associations between media use and mental health given decades of genetic research demonstrating that measures of the environment are partly heritable. As such, it is unclear whether shared genetic influences partly confound the relationships between media use and mental health. This could happen, for example, if heritable traits such as impulsiveness influence social media use and confer risk to mental health problems (e.g., ADHD). This research answers the call for novel approaches to address the role of individual differences in interactions with digital technologies^28^.

To date, a limited number of genetically informed studies have examined the relationship between media use and mental health. These studies focussed primarily on the negative side of online media use, including online bullying, victimisation and problematic internet use and were largely based on small twin samples^29–33^ or candidate gene studies^34^.

The present study investigates the links between explicitly negative *and* general media use and mental health in young people using multiple genetically informed approaches, with complementary strengths. In a large sample of British twins, we applied twin analyses and polygenic scoring methods to (1) estimate overall genetic influence on online media use and measures of mental health and wellbeing and (2) to assess possible genetic origins of the association between media and mental health. In addition, we employed a novel genetic sensitivity analysis to examine genetic confounding in the media use and mental health association with increasing polygenic score accuracy (to that of SNP and twin-based heritability estimates).These complementary analyses rely on different assumptions, enabling triangulation of evidence on the genetic origins of media use and its association with mental health.

## Materials and methods

### Sample

Analyses were based on participants in the Twins Early Development Study (TEDS), a longitudinal birth cohort study of twins born in England and Wales between 1994 and 1996. TEDS participants with available phenotypic data on measures of general media use (n = 3930 twin pairs), online victimisation (n = 3931 twin pairs) and problematic media use (n = 3934 twin pairs), were included in the present study. Sample sizes for all media and mental health measures by zygosity including the number of monozygotic (MZ), dizygotic same sex (DZss) and dizygotic opposite sex (DSoss) twin pairs can be found in Supplementary Tables S1 and S2. Despite some attrition, the remaining cohort, as well as the genotyped subsample have been shown to reasonably represent the UK population for that birth cohort (i.e., 93% white in National averages from UK cohorts of parents with children born in late 1990’s early 2000’s compared to 92.9% white in TEDS)^20^. DNA was obtained and genotyped for one member of a twin pair, yielding a genotyped sample of unrelated individuals with media and mental health measures for the DNA based analyses of 6415. Both the twin sample (92.9%) and the genotyped subsample (99.9%) were predominately white European, therefore findings cannot readily generalise to other populations. Ethical approval for this study was received from King’s College London Ethics Committee and all methods were carried out in accordance with the relevant guidelines and regulations. All participants provided written informed consent.

### Phenotypic measures

Phenotypic measures were collected when the twins had reached emerging adulthood (M age = 22.14, SD = 0.85)^20^. Data were collected using either a smartphone application, via the web, or paper and pencil questionnaires.

#### Problematic media use

A large literature demonstrates robust associations between problematic internet use and negative life outcomes including sleep disturbances, depression and poor social adjustment yet psychometrically validated screening instruments are lacking. We assessed problematic media use using an adapted version of the Problematic and Risky Internet Use Screening Scale^35^ (PRIUSS). This 18-item scale assessed problematic internet use that resulted in social or emotional impairment, including questions such as ‘How often have you lost motivation to do other things that need to get done because of the internet’ and ‘How often have you lost sleep due to nighttime internet use’. Based on analyses of pilot data, the scale was reduced to a 6-item measure with responses given on a 5-point scale (0 = “never”, 1 = “rarely”, 2 = “sometimes/quite often”, 3 = “often”, 4 = “very often/always”, 5 = “always”,). The 6 items included: ‘Have you felt irritated when the internet is not working?’, ‘Have you experienced feelings of withdrawal from not using the internet?’, ‘Have you prioritised internet use over important, everyday activities?’, ‘Have you lost motivation to do other things that need to get done because of the internet?’, ‘Have you lost sleep due to night time internet use?’, ‘Do you feel you have used the internet excessively?’. The PRIUSS shows excellent reliability and good validity, but was specifically developed to identify problems associated with Internet use. To better capture mobile phone use specifically, we amended the original questions, changing ‘internet’ to ‘on your mobile phone’. The ammended scale demonstrated high internal consistency (Cronbach’s alpha = 0.92) and good test–retest reliability (r = 0.70).

#### Online victimisation

Online media use in young adulthood coincides with a neurodevelopmental period of less self-regulation and heightened social and emotional vulnerability, as many mental health problems have their onset during this period. Increased online media use may expose some young people to negative consequences such as being the perpetrator or victim of bullying and represents an important aspect of online environments needing further genetically sensitive research attention. For this reason, we assessed online victimisation using the Multidimensional Peer Victimization Scale–Revised^36^ (MPVS-revised)which assessed the occurrence of online aggression and whether the individual was the target or perpetrator. Online victimisation frequently co-occurs with face-to-face bullying victimisation, but with the increased anonymity of the perpetrator and its inescapable nature, due to not being confined to particular locations or times (i.e., at school), may represent a potent threat to young people’s mental health. The MPVS-revised is a reliable measure with good psychometric properties and has been validated in a UK sample like the present study. For the present analyses, only items about online victimisation were included. Items assessing online victimisation included, for example, “How often has someone sent you a nasty text (excluding family or partner)?” and “How often has someone written something spiteful about you in a chat room (excluding family or partner)?”. Online victimisation was measured in four items, with responses given on a 3-point scale (0 = “not at all”, 1 = “once”, and 2 = “more than once”). These measures showed good reliability (Cronbach’s alpha = 0.89) and test–retest reliability (r = 0.79).

#### General media use

Current research trends tend to view media use as primarily problematic and negative for young people. A small but growing body of literature^37^ suggests that, for some people, online media use can be beneficial. For example, online media use might facilitate individualised learning, creating social networks, accessing online support, and maintaining relationships. We created a ‘general media use’ measure by adapting the Media and Technology Usage and Attitudes Scale (MTUAS)^38^. The MTUAS assessed media usage across 11 scales representing smartphone usage, general social media usage, Internet searching, e-mailing, media sharing, text messaging, video gaming, online friendships, Facebook friendships, phone calling, and watching television. An additional four subscales assessed attitudes toward media use: positive attitudes, negative attitudes, technological anxiety/dependence, and attitudes toward task-switching. Based on analysis of extensive pilot data, we reduced the MTUAS to 11 items capturing three media use scales including social media use, video game use and general phone use. These scales showed good reliability (Cronbach’s alpha = 0.8 and above) and test–retest reliability (r = 0.73 and above). We also created a composite of these three scales as a measure of general media use. The composite included 6 items assessed on a 6-point scale (1 = “Never”, 2 = “Several times a year”, 3 = “Several times a month”, 4 = “Several times a week”, 5 = “Several times a day”, 6 = “Several times an hour”). The items included in our reduced MTUAS scale are provided in Supplementary Table S3.

#### Mental health

Parent and self-report measures of mental health were included in the TEDS emerging adulthood assessment. The Strengths and Difficulties Questionnaire (SDQ), a 25-item assessment of positive and negative behavioural attributes, was used to assess five broad domains including emotional symptoms, conduct problems, hyperactivity/inattention, peer relationship problems, and prosocial behaviour^39^. The Cronbach’s alphas for each of the subdomains suggest good reliability (emotional symptoms = 0.67, conduct problems = 0.64, hyperactivity = 0.71, peer problems = 0.61, prosocial behaviour = 0.70), with good test–retest reliability (ranging from 0.70 to 80). In addition, the first four domains (excluding prosocial behaviour) were summed to create a total behavioural problems score. We considered the prosocial behaviour subdomain of the SDQ as an indicator of positive mental health, which is in line with previous research indicating prosociality correlates with markers of wellbeing, life satisfaction and happiness.

The Moods and Feelings Questionnaire (MFQ) is a screening tool used to assess DSM-III-R criteria for depression in children and young people^40^. The MFQ includes a series of descriptive phrases assessing how the individual has been feeling or acting recently, for example: ‘I felt I was no good anymore’; ‘I felt lonely’; ‘I hated myself’. A total score taking the mean of the 11 MFQ items was derived for the purposes of these analyses. The MFQ showed good reliability (Cronbach’s alpha = 0.91) and test–retest reliability (r = 0.75).

Sample sizes for the media and mental health measures are detailed in Supplementary Online Table S1.

#### Genome-wide polygenic scores

Nine genome-wide polygenic scores (GPS) indexing psychiatric disorders and associated traits (i.e., education), were created in our independent sample of unrelated individuals based on publicly available genome-wide association (GWA) summary statistics from the Psychiatric Genetics Consortium: Schizophrenia^41^, Bipolar Disorder^42^, Major Depressive Disorder^43^, Autism Spectrum Disorder^44^, Attention-Deficit/Hyperactivity Disorder^45^, Obsessive–Compulsive Disorder^46^, Anorexia Nervosa^47^, Post-Traumatic Stress Disorder^48^; and the Social Science Genetic Association Consortium: Years of Education^49^ (Supplementary Table S4). Construction of the GPS is described in detail in the Supplementary Online Methods.

## Statistical analyses

### Phenotypic analyses

To examine the overall associations between media use and mental health, we computed correlations between each of our media use and mental health measures. The p-values for the correlations were adjusted for multiple comparisons using the false discovery rate (FDR) method^50^. Raw values for the phenotypic correlations between all media and mental health measures, separately for girls and boys, are reported in Supplementary Fig. S1a,b. Means and standard deviations for the media use measures are reported for the whole sample, males and females separately, and for all five sex and zygosity groups: monozygotic (MZ) males, dizygotic (DZ) males, MZ females, DZ females, and DZ opposite-sex twin pairs (Table S5). In order to maintain independence of data, one twin per pair was randomly selected, and analyses of variance (ANOVA) was performed to test the significance of these group differences. The ANOVA results indicated that sex and zygosity together account for less than 1% of the variance across measures, therefore to increase power all subsequent analyses were performed on the full sample, combining males and females and including opposite-sex pairs. All variables were age and sex regressed. In the analysis of twin data this age and sex regression is necessary because members of a twin pair are identical in age and MZ twins are also identical for sex, and this would otherwise inflate twin estimates of shared environment^51^. For the subsequent twin analyses, all measures were corrected for skewness by mapping them on to a standard normal distribution using the rank-based van der Waerden's transformation (vdW)^52,53^, analyses were compared with and without vDW and results were broadly consistent.

## Genetic analyses

### Twin analyses

#### Univariate twin analyses

To assess the relative genetic and environmental contributions to variance in general media use, online victimisation and problematic media use, we used univariate twin analyses. Heritability (A), refers to the genetic contribution to phenotypic variance and is narrowly defined as the proportion of individual differences in a population that can be attributed to additive effects of inherited DNA differences between individuals. Doubling the difference between MZ and DZ correlations on a trait provides a rough estimate of ‘A’. Environmental influences in twin analyses are split into those non-inherited influences that are shared (C) and unique (E) to twins growing up in the same home. C contributes to twin similarity and can be calculated by subtracting ‘A’ from the MZ twin correlations. The E component is calculated by deducting the A and C components from unity. In addition to capturing environmental experiences unique to the individual, E includes measurement error.

The ACE estimates and their confidence intervals can be calculated more precisely using structural equation modelling (SEM) with the OpenMX software package^54^.

#### Bivariate twin analyses

To investigate genetic and environmental covariance between online media use and mental health outcomes, we used bivariate twin analyses. Bivariate twin analyses are an extension of univariate twin analyses that rely on cross-twin cross-trait correlations to decompose phenotypic covariance between multiple traits into genetic and environmental components of covariance. We estimated the proportion of the phenotypic correlation between online media use and mental health measures that is explained by genetic (rphA), shared environmental (rphC), and nonshared environmental (rphE) influences. Detailed description of univariate and multivariate twin analyses in the TEDS sample have been published^55^.

#### Polygenic score analyses

To investigate the contribution of measured genetic liability to psychopathology and related traits to (1) media use, and (2) the association between media use and mental health, we applied multiple polygenic score analyses, which have been shown to increase predictive power when compared to single polygenic score approaches^56^. We conducted multiple regression analyses to investigate the proportion of variance in our media use measures that can be accounted for using polygenic scores (GPS) as well as the relative contribution of each of the 9 GPS to trait prediction. Beta coefficients were inspected to investigate the sign (positive or negative) of the effect—whether higher polygenic loading is associated with increased or decreased risk of general media use, online victimisation and problematic media use.

### Genetic sensitivity analysis

Current polygenic scores explain much less of the heritable variance in psychiatric traits than is expected from twin studies, in part due to their reliance on additive effects of common (present in more than 1% of the population) Single Nucleotide Polymorphisms (SNPs) and dependence on the sample size of the discovery GWAS for which their effect estimates are derived. As such, polygenic scores are likely to under-estimate genetic confounding. To address this, we employed a novel genetic sensitivity analysis called Gsens. Gsens involves estimating genetic confounding from a Structural Equation Model based on a matrix of observed correlations between polygenic scores, exposure and outcomes. Critically, this correlation matrix can be modified under scenarios in which polygenic scores explain greater heritability in the outcomes (by using estimates of SNP and twin-based heritability). This enables estimation of genetic confounding based on more predictive (latent) polygenic scores. Further methodological details are provided in. In line with recommendations, the sensitivity analysis was restricted to those mental health outcomes for which we had corresponding polygenic scores, namely emotional symptoms (for which we used a polygenic score for anxiety), depression (for which we used the depression polygenic score) and hyperactivity (for which we used the ADHD polygenic score). The SNP and twin heritability estimates used for the Gsens analyses are reported in Supplementary Table S7.

All statistical analyses were conducted in Linux environment using R version 3.5.0^57^.

## Results

### Phenotypic associations between media use and mental health

**Figure 1:**
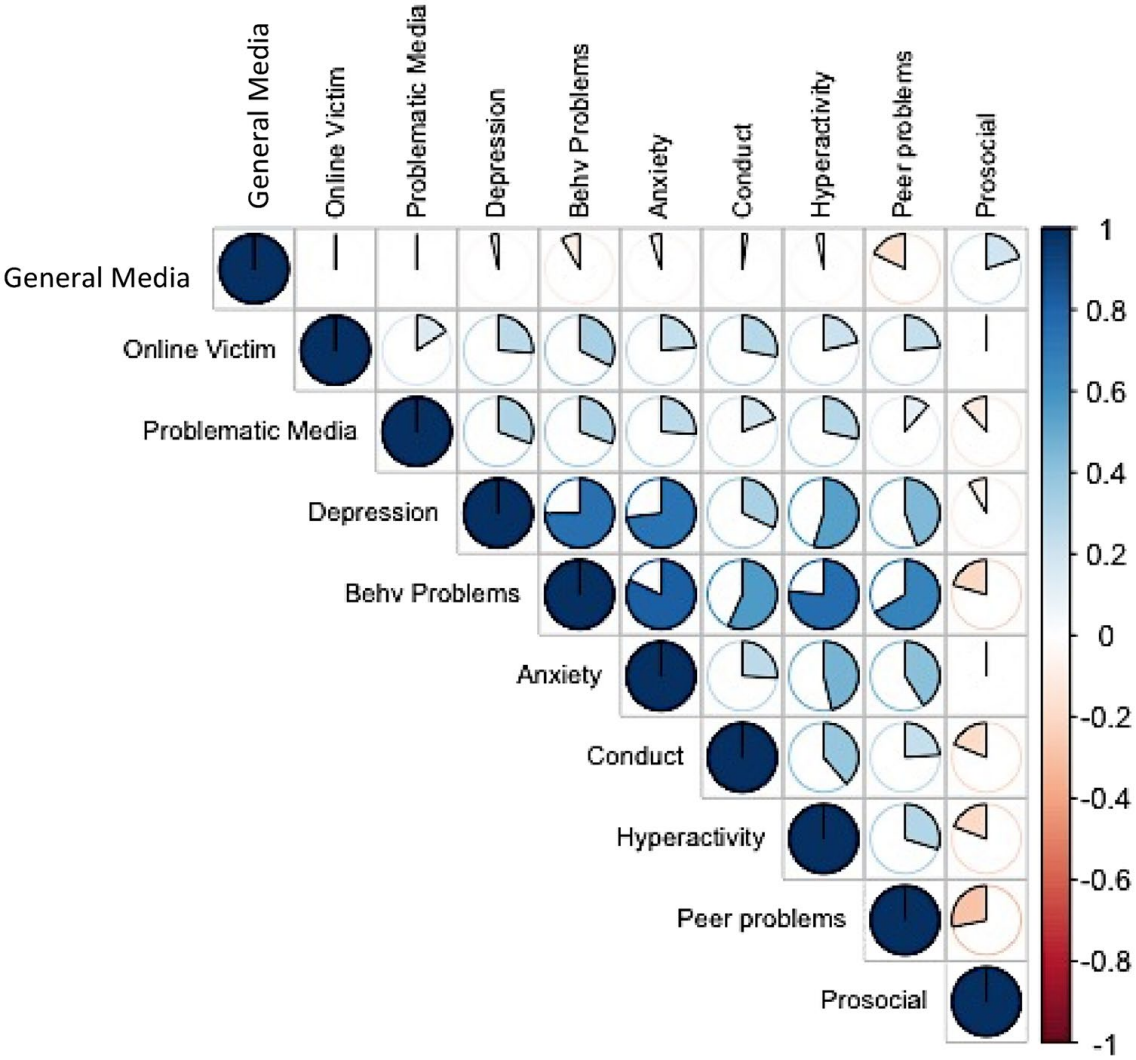
Phenotypic correlations between media use and mental health measures. *Note*: Behv Problems = the behavioural problems subdomain of the Strength and Difficulties Questionnaire^39^.

Figure 1 shows the phenotypic correlations between general and explicitly negative media use and each of our mental health measures. Negative media use (online victimisation, problematic media use) was associated with mental health problems, including depressive symptoms, behavioural and peer problems, anxiety symptoms, conduct behaviour and hyperactivity. Problematic media use was also associated with less prosocial behaviour. By contrast, general media use was modestly associated with fewer symptoms of mental health problems, with the most robust evidence emerging for fewer peer problems. General media use was associated with more prosocial behaviour. Results were similar for girls and boys (see Fig. S1a,b).

### Twin analyses

#### Genetic influences on media use

Results from our univariate twin analyses are illustrated in Table 1. Heritability estimates were 43% for problematic media use, 26% for online victimisation and 49% for general media use. We did not find evidence for shared environmental influences for any of the three measures of media use.

**Table 1:** Univariate twin analyses indicating the proportion of total variance in general media use, online victimisation and problematic media use captured by genetic and environmental sources of variation (95% confidence intervals in parentheses).

Media use	A	C	E	Intraclass correlations	
				MZ	DZ
Problematic media	0.43	0.00	0.57	0.46	0.17
	(0.40–0.45)	(0.00–0.00)	(0.54–0.60)	(0.43–0.49)	(0.14–0.20)
Online victimisation	0.26	0.00	0.74	0.26	0.15
	(0.23–0.30)	(0.00–0.05)	(0.70–0.77)	(0.23–0.29)	(0.12–0.18)
General media	0.49	0.00	0.51	0.49	0.23
	(0.42–0.51)	(0.00–0.00)	(0.49–0.54)	(0.46–0.51)	(0.21–0.26)

A = additive genetic factors; C = shared (common) environmental factors that make siblings growing up in the same home more similar; E = unique/nonshared environmental factors that do not correlate between siblings, including measurement error. Intraclass correlations are reported for monozygotic (MZ problematic media n = 2890, online victimisation n = 2888 and general media n = 2886) and dizygotic (DZ problematic media n = 4879, online victimisation n = 4878 and general media n = 4858) twin pairs, including DZ opposite sex pairs. The 95% confidence intervals are presented under each estimate in brackets.

#### Genetic influences on the associations between media use and mental health

**Figure 2:**
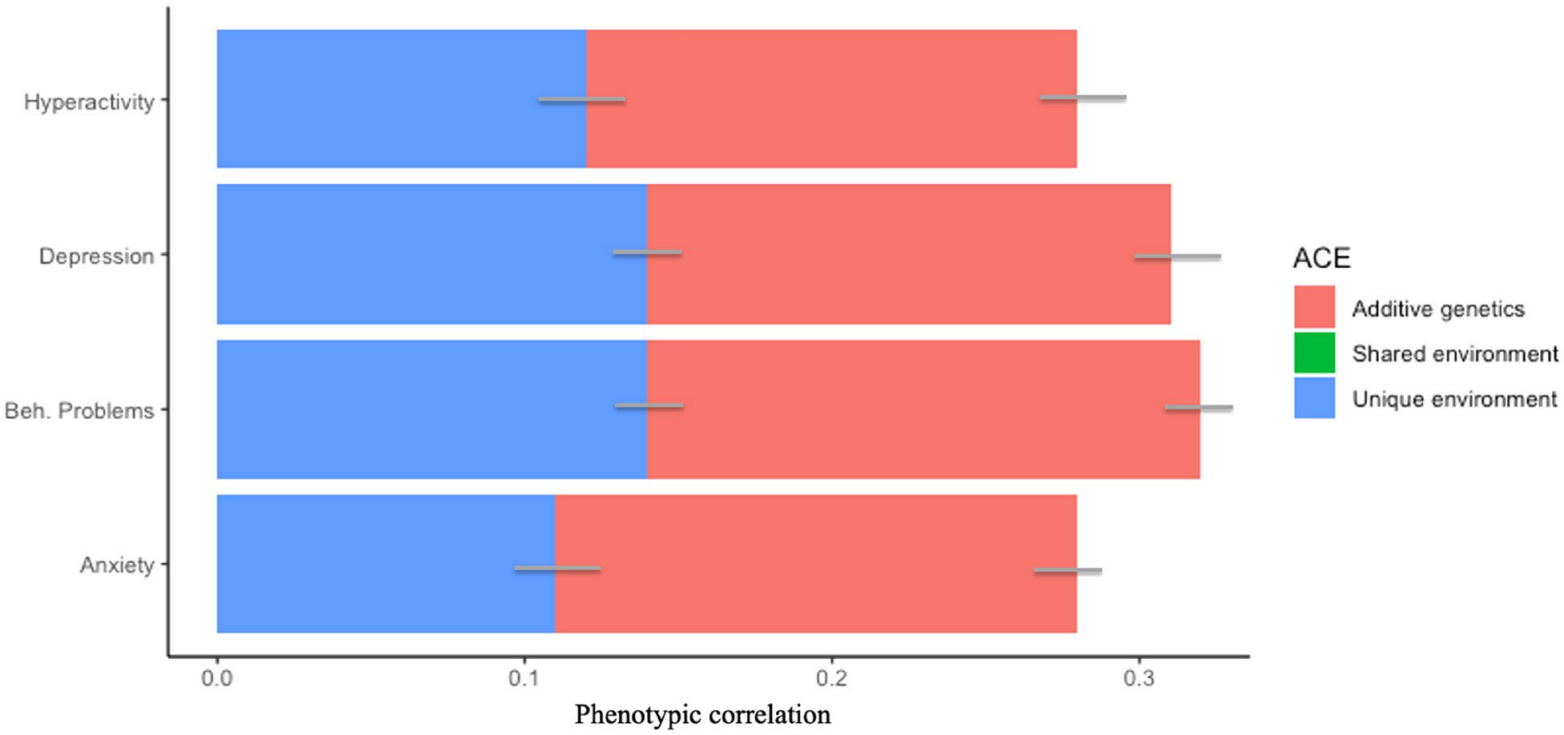
Genetic and environmental contribution to phenotypic associations between problematic media use and mental health. *Note*: Bars represent 95% confidence intervals around estimates. Hyperactivity = total hyperactivity score derived from the subscale of the Strength and Difficulties Questionnaire (SDQ)^39^, Depression = depressive symptom scores measured by the Mood and Feelings Questionnaire (MFQ)^58^; Beh. Problems = total behavioural problems score derived from the subscale of the SDQ^39^.

**Figure 3:**
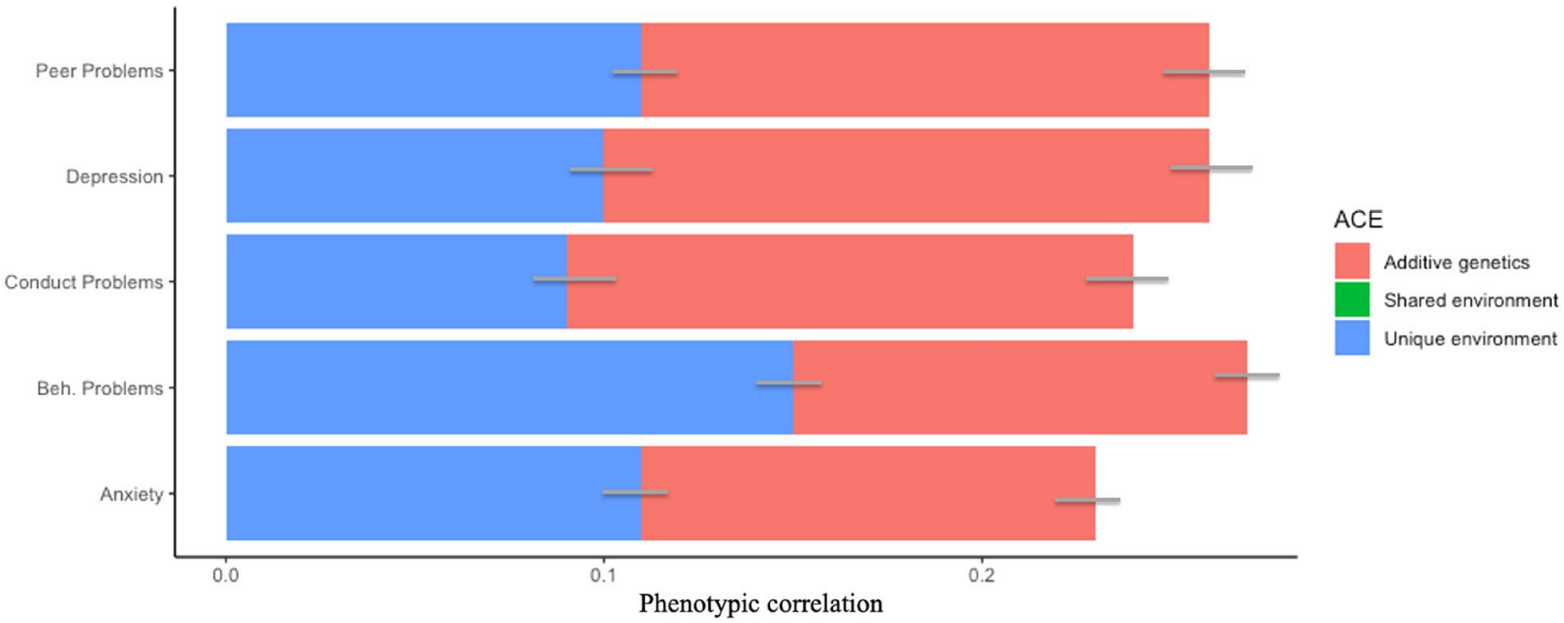
Genetic and environmental contribution to phenotypic associations between online victimisation and mental health. *Note*: Bars represent 95% confidence intervals around estimates. H Peer = total peer problems score derived from the subscale of the Strength and Difficulties Questionnaire (SDQ)^39^, Depression = depressive symptom scores measured by the Mood and Feelings Questionnaire (MFQ)^58^; Conduct Problems = total conduct disorder symptoms derived from the subscale of the SDQ^39^. Beh. Problems = total behavioural problems score derived from the subscale of the SDQ^39^. Anxiety = total anxiety score derived from the subscale of the SDQ^39^.

**Figure 4:**
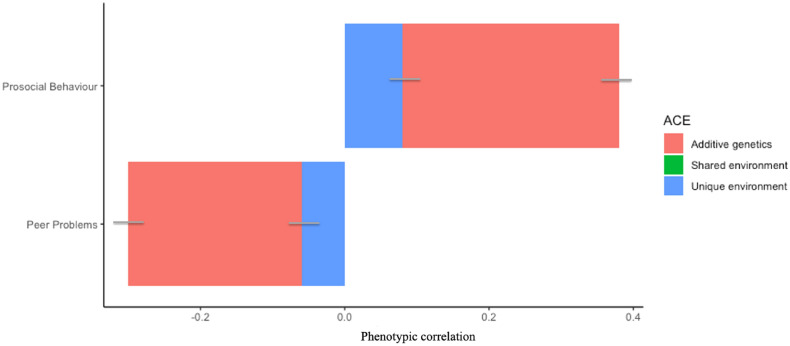
Genetic and environmental contribution to phenotypic associations between general media use and mental health. *Note*: Bars represent 95% confidence intervals around estimates. Prosocial Behaviour = prosocial behaviour score derived from the subscale of the Strength and Difficulties Questionnaire (SDQ)^39^, Peer Problems = total peer problems score derived from the subscale of the SDQ^39^.

We computed bivariate twin analyses to estimate the proportion of the correlations between online media use and mental health that was driven by genetic and environmental factors (Figs. 2, 3, 4).

For those media use measures that demonstrated robust (r ≤ 0.20) phenotypic associations with mental health measures, a large part of their association was accounted for by additive genetic factors (ranging from 38% [online victimisation and conduct problems] to 88% [general media use and prosocial behaviour]). Shared environmental factors did not contribute to the association between media use and mental health for problematic media use, online victimisation or general media use (Figs. 2, 3, 4). The bivariate twin estimates and confidence intervals are detailed in Supplementary Table S6.

#### Genome-wide polygenic score results

Genome-wide polygenic score (GPS) analyses also indicated genetic influence on both general and explicitly negative media use, although effect sizes were small, explaining less than 1% of the variance. For problematic media use, of the 9 tested GPS, higher schizophrenia GPS (r = 0.057, *p* < .001), higher ASD GPS (r = 0.038, *p* = .01) and lower education GPS (r =  − 0.055, *p* < .01) associations survived multiple testing correction. Online victimisation was associated with four GPS, including higher MDD GPS (r = 0.036, *p* = .01), higher PTSD GPS (r = 0.049, *p* < .001), higher ADHD GPS (r = 0.065, *p* < .001) and lower education GPS (r =  − 0.084, *p* < .001). Finally, for general media use, three significant GPS associations emerged including lower schizophrenia GPS (r =  − 0.043, *p* < .001), lower ASD GPS (r =  − 0.040, *p* < .001), and higher education GPS (r = 0.047, *p* < .001). Together, the 9 GPS yielded significant (*p* < .001) multiple correlations with problematic media use (R = 0.079), online victimisation (R = 0.099) and general media use (R = 0.066). Correlations between each of the 9 GPS and the media use measures are presented in Supplementary Fig. S2.

### Sensitivity analyses results

Results from our Gsens analysis using polygenic scores to test for genetic confounding on the association between media use and mental health are provided in Figs. S3–S18 of the Supplementary Online Material. For consistency with our twin analyses, Gsens was performed on media use and mental health measures where phenotypic correlations were ≥ 0.20).

Effect sizes of our media use and mental health associations were reduced by 0.6–1.8% using the observed depression, anxiety and ADHD polygenic scores, indicating little genetic confounding (Figs. S3, S6, S9, S12, S15).

We then tested for genetic confounding under a SNP- and twin- based scenario where polygenic scores explain the upper bound SNP- and twin- based heritability of mental health measures, respectively (Figs. S4, S7, S10, S13, S16). The results show that under a SNP heritability scenario, the association between problematic media use and mental health is attenuated by 23–100%. A similar pattern of results was observed for online victimization where under the SNP based scenario effect sizes for the association with mental health were decreased by 41–81%.

For both problematic media use and online victimization, under a twin-based scenario where polygenic scores explain twin heritability, the media use and mental health association was completely confounded by genetics (Figs. S5, S8, S11, S14, S17).

**Figure 5:**
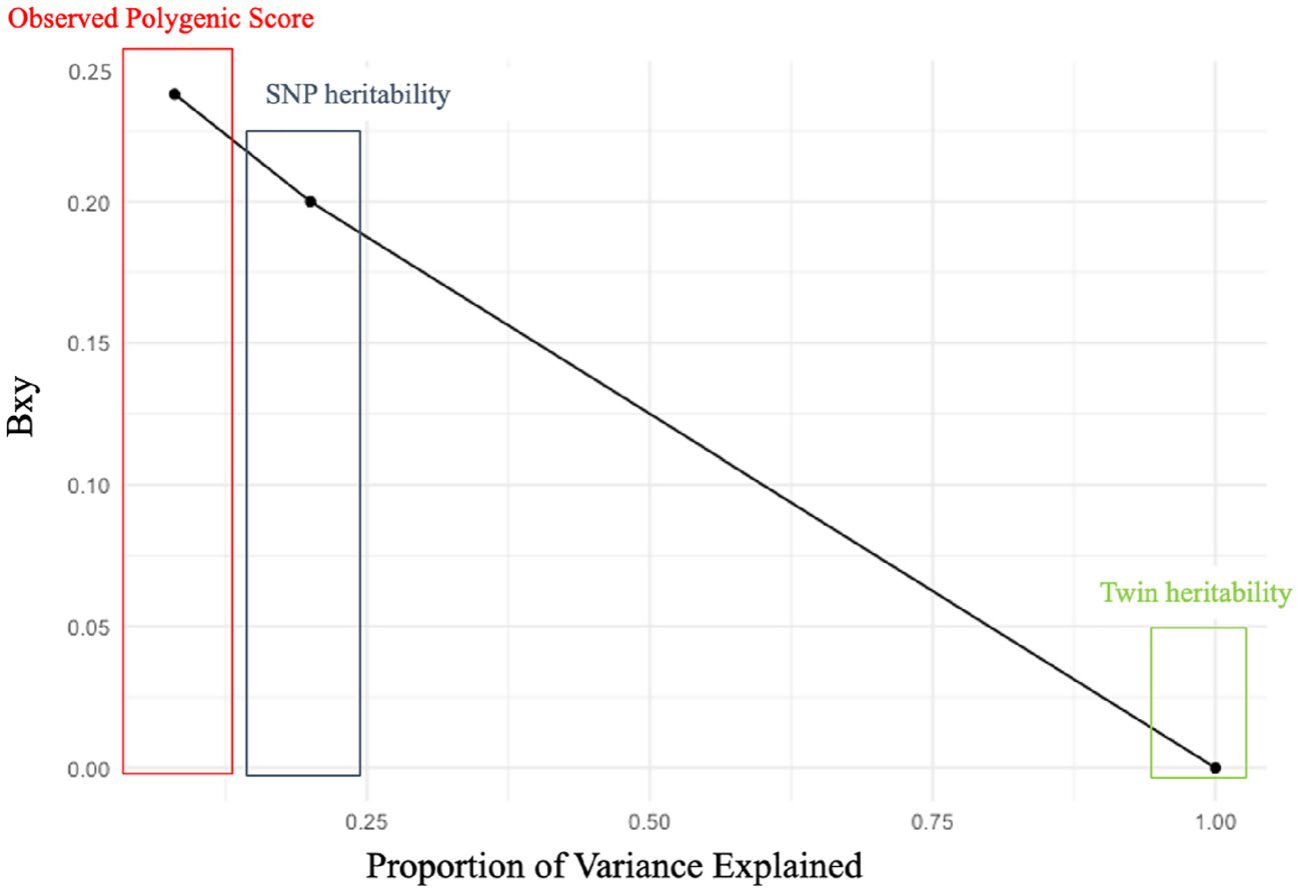
Genetic confounding in the association between online victimization and depressive symptoms when adjusting for observed polygenic scores (red), and under a SNP (blue) and twin (green) heritability scenario. *Note*: Bxy = standardized estimate of the relationship between online victimisation and depressive symptoms when using observed depression polygenic scores (red), depression polygenic scores that explain SNP heritability in depression (blue) and depression polygenic scores that explain twin heritability in depression (green).

For simplicity, we only depict Gsens results for the association between online victimisation and depression below (Fig. 5) and refer the reader to supplementary for the remainder of the Gsens results, including full model output (Supplementary Fig. S18).

## Discussion

Extant genetic research on the impact of online media use on mental health has predominantly focused on the deleterious effects of excessive media use. In a sample of nearly 8000 emerging adults, we demonstrate that general media use correlates phenotypically with fewer peer problems (r =  − 0.15) and more prosocial behaviour (r = 0.19), a measure of positive mental health. Capitalising on approximately 4000 twin pairs we have demonstrated that genetic influences (1) affect both the general and explicitly negative sides of online media use, and (2) partly underlie the associations between online media use and mental health. Indeed, bivariate twin analyses on all robust phenotypic correlations indicate that 38 to 62% of the correlation between negative media use and negative mental health and 80 to 88% of the correlation between general media use and positive mental health is driven by genetic influences. Genome-wide polygenic score (GPS) analyses also indicate genetic confounding in the association between media use and mental health, explaining up to 100% of the association with increasing polygenic score accuracy.

Finding genetic influences on associations between mental health and online media use in emerging adulthood confirms and extends previous analyses using the same sample where we demonstrated genetic influence on home computer use when the twins were aged 16^19^. Importantly, here we found strong evidence that the associations between media use and mental health problems are specific to negative uses of the media including experiences of online victimisation and problematic internet use. These results represent an important first step in better characterising media use and mental health associations and highlight the need for further genetically informed analyses. Inclusion of longitudinal designs and causal inference models such as Mendelian Randomization will be essential to better understanding the temporal causality of these associations while more adequately accounting for confounding^59^. For example, our finding of genetic contributions to media use and mental health associations does not preclude reciprocal causal influences between media use and mental health.

Genetic differences contribute to how young people create their online environments and support the widely demonstrated finding that many so-called “environments” are themselves under substantial genetic influence^60,61^. Shared environmental influences, which include those factors that make siblings growing up in the same home more similar, were zero for the media use measures, which may in part reflect the fact that these young people have now left their family home and therefore parental monitoring on media use is less prominent. Polygenic score analyses indicated that young people with genetic liability to underlying mental health problems may be particularly vulnerable to using online media use in a negative way, which may in turn further impact their wellbeing at a time when they have less familial support. Finding no shared environmental effects in young adulthood is consistent with decades of behavioural genetic research which demonstrates that genetics, and not the shared environment, contributes to similarities between family members post-childhood. This interpretation can explain why for example, parents and their children can show high correlations in their media use, because parents and their offspring share on average 50% of their genetic material.

The absence of shared environmental influence also reflects the consistent behavioural genetic finding that when individuals are increasingly free to select their environments, we see greater genetic influences while shared environmental influences decrease developmentally^11^. Because young adults have greater choice and autonomy over their media use compared to teenagers, genetic influences manifest more strongly while the influence of the shared family environment decreases. We observe this trend when we compare the current results to our previous analyses on genetic influence on media use on the home computer when the twins were aged 16. Shared environmental influences were significant (8–20%) for home computer use in their teens compared to now when they are young adults freer to create, modify and interpret their online experiences.

Co-occurring rises in online media use and self-reported mental health problems in early adulthood have garnered considerable research attention^27^. The complexity of these relationships within the rapidly evolving media landscape requires novel approaches if we are to better understand individual differences and their impact. Greater nuance is needed within these investigations, including a more balanced view of the potential positive impacts of media use, which we sought to incorporate in the present study by including a general media use measure. More interdisciplinary approaches are needed to better understand the nature of media use—and as we demonstrate here, with sensitivity to genetic contributions.

Importantly these results indicate the cooccurrence of media use and mental health problems can be largely accounted for by common genetic vulnerabilities. This suggests that the inherited characteristics that make individuals more vulnerable to mental health problems are also increasing their vulnerability to online victimisation and for example, loss of sleep due to night-time internet use.

This was suggested not only by the bivariate twin results, but also by the genetic sensitivity analysis, which estimated genetic confounding under scenarios in which polygenic scores captured increasing heritability in the outcomes. Under a scenario in which polygenic scores captured SNP heritability in mental health outcomes, the associations between media use and mental health were attenuated by at least 20%. However, when accounting for greater genetic variance using twin heritability estimates, the associations between media use and mental health were attenuated to null. Future studies could employ these methods to explore, for example, how other mental health symptoms relate to media use, even with the current limited predictive power of psychiatric polygenic scores.

Although media use is ubiquitous in early adulthood, experiences and uses differ and are not always negative. For example, there is a growing body of literature showing that for marginalized communities, social media may in some circumstances serve as a platform to share resources and curate self-affirming content. These aspects of media use are understudied in general, and especially in genetically informed designs where most samples are obtained from white European samples. This is a key limitation in the present study as the TEDS twin (93%) and genotyped sample (99%) is predominately white. Until studies better represent global diversity, our understanding of media use will be inadequate^27^ and work will continue to perpetuate disparities in health research with knowledge benefits largely withheld from racialised communities^62,63^.

Our results must be understood within the context of the known limitations of the twin and polygenic score designs^64^. Discrepancy between twin and polygenic score-based estimates of genetic influence are expected given unlike twin studies which capture *all* forms of genetic variation including gene–gene and gene-environment interactions, polygenic scores are limited to the additive effects of common SNPs. Our genome-wide polygenic scores are not subject to the same assumptions as the twin method. However, our GPS analyses are limited to the power of current genome-wide association studies (GWAS), the size of their discovery samples and the biological validity of current diagnostic systems^65^. Triangulation of our results using these complementary genetic methods with different assumptions provides further evidence for the need for genetically informed designs to better understand the reciprocal causal relationships between those phenotypes.

As TEDS is a population representative sample, severe experiences of behavioural and mental health problems may be less represented, although behavioural genetic evidence indicates that common disorders represent the extreme end of a behavioural continuum and therefore our population sample can still provide useful insights to the aetiology of these associations^66^.

Our findings should also be considered in the context of the measures used. All the media use and mental health measures were self-reported, and therefore rater bias may have influenced our results^67^. Indeed, previous studies have found that the associations between media use and mental health are stronger for self-reported outcomes compared to outcomes reported by other informants^26,68,69^. This is a concern with all self-report measures and efforts are needed to include multiple sources of information from different raters (self, parent, teacher) and in ancestrally diverse samples. In addition, for the purposes of the present study and to capture variability in mental health, we considered prosocial behaviour as a marker of positive mental health. While there is a literature supporting this interpretation, we acknowledge that the relationship is likely more complex, with some evidence for a ‘goldilocks’ effect’ whereby too much or too little prosocial behaviour is associated with poor mental health.

We highlight and acknowledge the shortcomings in our measure of general media use, which was derived from the residuals of a regression of the general media use composite on the available problematic media use and online victimisation scales. Our general media use measure correlated highly with the composite derived from the validated Media and Technology Usage and Attitudes Scale phenotypically and genetically. We chose this approach to steer future research toward addressing the clear gap in diverse media experiences beyond explicitly negative use, however we strongly encourage new development of questionnaires designed to interrogate ways in which young people find media use positive. Large, genotyped cohort samples of representative young adults have great potential for investigating the role of positive and general media use in mental health. Although we were limited in the media use measures that are currently available in TEDS, we hope that this research can spur discussion in other cohorts to collect broader measures^70,71^. Critically, scales are needed that go beyond measuring amount of time spent on social media devices to query the psychological processes, motivations, and socialising goals that help to sustain these behaviours. Distinctions between active, passive, social and non-social media use are an important nuance in how we define future scales as well as the need to integrate knowledge from focus groups and in-depth discussion with young people about their ever-changing experiences online . Despite this limitation, our results indicating an association between general media use and positive mental health outcomes is consistent with a recent study where routine social media use as measured by an adapted general media use scale was associated with social well-being, positive mental health, and self-rated health.

Recent valid criticisms that many of the scales used to assess problematic media use have been raised. These include concerns that (1) in lumping diverse measures of media use together researchers may be grouping constructs that originate from very heterogenous personal characteristics with distinct etiological mechanisms (2) we are pathologizing normal behavior (3) in the eagerness to measure online behavior less emphasis has been placed on validating the psychometric properties of the scales used. These limitations can be uniquely addressed using genetics. Diverse measures of media use can be tested for their underlying genetic structure and in doing so can isolate constructs of similar etiologies. Twin and DNA based methods can be used to compare the heritability of diverse measures of media use with the expectation that those scales more reliably tapping into true variation in online behavior also display the highest contribution by additive genetic variation. Combining longitudinal approaches with genetics can help provide nuance to item selection as variation in the problematic aspects of media use is likely more complex than the simplistic ‘more is worse’ approach currently adopted. Despite the replication crisis in science, it is noteworthy that genetic and genomic results replicate reliably. For this reason, despite the limitations of current scales, it is not the time to throw the baby out with the bathwater, but instead to capitalize on genomics as a tool to inform our understanding of these complex online environments and to construct better, more reliable tools for advancing knowledge of this ubiquitous aspect of young adulthood.

In our increasingly digital word, young adults are disproportionately using digital devices and increasingly reporting mental health problems. Our findings show that observed associations between media use and mental health are largely accounted for by genetic influences. As such, future research efforts to understand the mental health impact of media use should adopt genetically informed designs to strengthen causal inference.

## Supplementary Information

Supplementary Information.

## Data Availability

Please refer to the Twins Early Development Study data access policy available here: https://www.teds.ac.uk/researchers/teds-data-access-policy.
