# Author Correction: Gene-environment correlations and genetic confounding underlying the association between media use and mental health

**DOI:** 10.1038/s41598-023-30112-1

**Published:** 2023-02-21

**Authors:** Ziada Ayorech, Jessie R. Baldwin, Jean-Baptiste Pingault, Kaili Rimfeld, Robert Plomin

**Affiliations:** 1 Social, Genetic and Developmental Psychiatry Centre, Institute of Psychiatry, Psychology and Neuroscience, King’s College London, London, SE5 8AF UK; 2 Department of Psychology, PROMENTA Research Center, University of Oslo, Oslo, Norway; 3 Department of Clinical, Educational and Health Psychology, University College London, London, WC1H 0AP UK; 4 Department of Psychology, Royal Holloway University of London, London, TW20 0EX UK

Correction to: *Scientific Reports* https://doi.org/10.1038/s41598-022-25374-0, published online 19 January 2023

The original version of this Article contained an error in Reference 68, which was incorrectly given as:

Baldwin, J., Rijsdijk, F., Schoeler, T., Arsenault, L., Ayorech, Z. & Pingault, J. Cyber-victimisation and mental health in young people: A co-twin control study. *PNAS*

The correct reference is listed below:

Baldwin, J. R., Ayorech, Z., Rijsdijk, F. V., Schoeler, T., & Pingault, J. B. Cyber-victimisation and mental health in young people: A co-twin control study. *Psychol. Med.* **51**(15), 2620–2630. https://doi.org/10.1017/S0033291720001178 (2021).

The original Article has been corrected.

