# Supplementary material for: Gene-environment correlations and genetic confounding underlying the association between media use and mental health
Source: Sci Rep. 2023 Jan 19;13:1030. doi: 10.1038/s41598-022-25374-0 (PMC9852440; doi:10.1038/s41598-022-25374-0)
Supplement: Supplementary file 1 — Supplementary Information. [file 41598_2022_25374_MOESM1_ESM.docx]

Supplementary Online Material

**Title**

Gene-environment correlations and genetic confounding underlying the association between media use and mental health

**Authors**

Ziada Ayorech^1,2,*^, Jessie R. Baldwin^3^, Jean-Baptiste Pingault^3^, Kaili Rimfeld^1,4^, Robert Plomin^1,^

**Author affiliations**

^1^ King's College London, Social, Genetic and Developmental Psychiatry Centre, Institute of Psychiatry, Psychology & Neuroscience, London, SE5 8AF, UK

^2^ PROMENTA Research Center, Department of Psychology, University of Oslo, Norway

^3^ Department of Clinical, Educational and Health Psychology, University College London, WC1H0AP, United Kingdom

^4^Department of Psychology, Royal Holloway University of London, TW20 0EX, United Kingdom

*Correspondence: Ziada Ayorech, PROMENTA Research Center, Department of Psychology, University of Oslo, Norway

Email: [ziada.ayorech@psykologio.uio.no](mailto:ziada.ayorech@psykologio.uio.no)

**Supplementary Methods**

Methods S1. Genotyping and Quality Control

Methods S2. Creating polygenic scores using LDpred

**Supplementary Tables**

Table S1. Sample sizes for all variables included in analyses including twin pairs and individuals

Table S2. Number of monozygotic (MZ) and dizygotic opposite sex (DZos) and dizygotic same sez (DZss) twin pairs included in analyses

Table S3. Items included in the reduced Media and Technology Uses and Attitudes Scale (MTUAS)

Table S4. Publicly available Psychiatric Genetics Consortium Genome-Wide Association studies used for creation of polygenic scores

Table S5. Means and standard deviations for the media use measure by the five twin groups arising from sex and zygosity

Table S6. Bivariate twin analyses for media use and mental health phenotypic correlations

Table S7. Single Nucleotide Polymorphism (SNP) heritability and twin heritability estimates used for genetic sensitivity analyses (Gsens)

**Supplementary Figures**

Figure S1a. Nominal values for the phenotypic correlations between mental health measures and measures of online media use in girls only

Figure S1b. Nominal values for the phenotypic correlations between mental health measures and measures of online media use in boys only

Figure S2. Genome-wide polygenic score (GPS) correlations between all 9 GPS and measures of online media use

Figure S3 -S18. Results from Gsens sensitivity analyses depicting genetic confounding on the media use and mental health with increasing accuracy of polygenic scores

Figure S18. Full model output for Gsens sensitivity analyses depicting genetic confounding on the media use and mental health with increasing accuracy of polygenic scores

**Supplementary Methods**

**Methods S1. Genotyping and Quality Control**

DNA from 8,122 individuals was extracted from saliva and buccal cheek swab samples and hybridized to HumanOmniExpressExome-8v1.2 genotyping arrays at the Institute of Psychiatry, Psychology and Neuroscience Genomics & Biomarker Core Facility. Raw image data were pre-processed in GenomeStudio according to Illumina Exome Chip SOP v1.4. (<http://confluence.brc.iop.kcl.ac.uk:8090/display/PUB/Production+Version%3A+Illumina+Exome+Chip+SOP+v1.4>) . Prior to genotype calling, 919 multimapping SNPs and 501 samples with call rate <0.95 were removed. Following initial QC, the program ZCALL was used to augment genotype calling.

DNA from 3,747 individuals was extracted from buccal cheek swabs and genotyped at Affymetrix, Santa Clara, California, USA. From the extracted DNA samples, 3,665 samples were successfully hybridized to AffymetrixGeneChip 6.0 SNP genotyping arrays ([http://www.affymetrix.com/support/technical/datasheets/genomewide_snp6_datashe et.pdf](http://www.affymetrix.com/support/technical/datasheets/genomewide_snp6_datashe%20et.pdf)) using experimental protocols recommended by the manufacturer.

Raw image data were pre-processed at the Wellcome Trust Sanger Institute, Hinxton, UK for genotyping as part of the Wellcome Trust Case Control Consortium 2 (<https://www.wtccc.org.uk/ccc2/>). All pre-processing was conducted according to the manufacturer’s guidelines (http://www.affymetrix.com/support/downloads/manuals/genomewidesnp6_manual.pdf). Following initial QC, the program CHIAMO was used for genotype calling (https://mathgen.stats.ox.ac.uk/genetics_software/chiamo/chiamo.html).

After initial quality control, the same quality control was performed on samples from each of the platforms (Illumina and Affymetrix) separately using PLINK (1), R (2) and BCFtools (3) and EIGENSOFT(4,5).

DNA samples were excluded from subsequent analyses on the basis of call rate (<0.98) suspected non-European ancestry, the presence of severe medical or psychiatry problems or severe medical complications during early gestation and relatedness other than dizygotic twin status. SNPs were excluded if the minor allele frequency was <0.5%, if more than 2% of genotype data were missing, or if the Hardy Weinberg *p*-value was lower than 10^-5^. Non-autosomal markers and indels were also removed. Association between SNP and the platform, batch, plate or well on which samples were genotyped was calculated; SNPs with an effect p-value < 10-4 were excluded.

A total sample of 10,346 samples, including 7,026 unrelated individuals from which 3,320 individuals had a genotyped dizygotic co-twin remained. Genotype data following quality control was available for 4,776 individuals and 559,772 SNPs from the illumine array and 2,250 individuals and 635,269 SNPs from the Affymetrix array.

Genomewide genotypes from the two arrays were separately phased using EAGLE2 (6) and imputed using the Haplotype Reference Consortium(7) using the Positional Burrows-Wheeler Transform method (8) and the imputation software Minimac3 1.0.13 (9), which are available from the Michigan Imputation Server (https://imputationserver .sph.umich.edu). A series of quality checks were performed before merging data from the two arrays and variants with info <0.75 were excluded and SNPs that were non-overlapping between platforms were removed.

After merging, minor allele frequency differences were tested for between platforms and SNPS with an effect p-value <10^-4^ were removed. Those SNPs with a Hardy-Weinberg p-value >10^-5^ were also removed. Following these criteria, 7,363,646 genotyped and well-imputed SNPs were retained for analyses. Only unrelated individuals were included in the present analyses. To ease high computational demands by the software LDpred (10) for polygenic scoring in large samples, we further excluded SNPs with info <1, leaving 515,100 SNPs for analysis.

**Methods S2. Creating polygenic scores using LDpred**

Genome wide polygenic scores were calculated using the Bayesian approach, LDpred, which has been shown to outperform predictive accuracy of the conventional clumping and p-value thresholding approach (10). Here, a posterior effect size is derived for each SNP by re-weighting the original summary statistic coefficient by the relative influence of a SNP given its level of linkage disequilibrium with surrounding SNPS and a prior on the effect size of each SNP. The prior is based on the heritability of the trait and the fraction of markers assumed to casually influence the trait. GPS is then calculated as the sum of the trait-increasing alleles weighted by their posterior effect size estimate. Unlike the conventional clumping and thresholding approach, LDpred retains all SNPs common between GWA summary statistics and genotype data in the target sample.

For the present study we applied a causal fraction of 1, which assumes that all SNPs contribute to the development of the trait. Due to the high computational demand of LDpred, especially in larger sample sizes including many SNPs, we made further restrictions to our analyses, only including the 515, 100 SNPs that were perfectly imputed (info score of 1) to reduce analytical load. Only genotypes of unrelated individuals were used to estimate LD structure in our sample because levels of LD are considerably higher in relatives compared to unrelated individuals (11).

**Supplementary Tables**

**Table S1.** Sample sizes for all variables included in analyses including twin pairs and individuals

| **Variable** | **N Twin Pairs** | **N Individuals** |
| --- | --- | --- |
| General Media use | 3,930 | 7,834 |
| Online victimisation | 3,931 | 7,836 |
| Problematic Media | 3,934 | 7,842 |
| SDQ anxiety | 4,088 | 8,141 |
| SDQ hyperactivity | 4,087 | 8,139 |
| SDQ conscientiousness | 4,088 | 8,144 |
| SDQ peer problems | 4,087 | 8,140 |
| SDQ prosocial | 4,088 | 8,147 |
| SDQ total problems | 4,088 | 8,141 |
| MFQ total problems | 4,080 | 8,123 |

**Table S2.** Number of monozygotic (MZ) and dizygotic opposite sex (DZos) and dizygotic same sez (DZss) twin pairs included in analyses

| **Variable** | **MZ** | **DZss** | **DZos** |
| --- | --- | --- | --- |
| General Media use | 2,886 | 2,487 | 2,371 |
| Online victimisation | 2,888 | 2,502 | 2,376 |
| Problematic Media | 2,890 | 2,503 | 2,376 |
| SDQ anxiety | 2,985 | 2,602 | 2,483 |
| SDQ hyperactivity | 2,985 | 2,601 | 2,482 |
| SDQ conscientiousness | 2,985 | 2,602 | 2,486 |
| SDQ peer problems | 3,104 | 2,728 | 2,624 |
| SDQ prosocial | 2,987 | 2,602 | 2,486 |
| SDQ total problems | 2,985 | 2,602 | 2,483 |
| MFQ total problems | 2,979 | 2,597 | 2,478 |

**Table S3.** Items included in the reduced Media and Technology Uses and Attitudes Scale (MTUAS)

| **Items included following extensive piloting of the original MTUAS* scale** |
| --- |
| *How often do you …* |
| Send, receive and read e-mails? |
| Send and receive text messages or check for text messages? |
| Make and receive calls on your mobile phone? |
| Check for voice calls? |
| Get directions online or use GPS? |
| Watch video clips? |
| Play games by yourself, with other people in the same room, or with other people online? |
| Check your social media account (such as Facebook, Instagram, Twitter, etc)? |
| Post status updates or photos? |
| Read posts by others? |
| Comment or click 'like' on postings, status updates, photos, etc? |

*Note:* *Rosen, L. D., Whaling, K., Carrier, L. M., Cheever, N. A., & Rokkum, J. (2013). The media and technology usage and attitudes scale: An empirical investigation. Computers in human behavior, 29(6), 2501-2511

**Table S4**. Publicly available Psychiatric Genetics Consortium Genome-Wide Association studies used for creation of polygenic scores

| **Trait** | **Cases** | **Controls** | **Sample** | **Year** | **PMID** |  |
| --- | --- | --- | --- | --- | --- | --- |
| SCZ | 40675 | 64643 | 105318 | 2018 | 29483656 |  |
| BIP | 7481 | 9250 | 16731 | 2011 | 21926972 |  |
| MDD^+^ | 59851 | 113154 | 173005 | 2018 | 29700475 |  |
| ASD | 18381 | 27381 | 46350 | 2017 | 30804558 |  |
| ADHD | 20183 | 35191 | 55374 | 2017 | 30478444 |  |
| OCD | 2688 | 7037 | 9725 | 2017 | 28761083 |  |
| AN | 3495 | 10982 | 14477 | 2017 | 28494655 |  |
| PTSD | 2424 | 7113 | 9537 | 2017 | 28439101 |  |
| EA3 | - | - | 1.1 million | 2019 | 30038396 |  |

**Table S5**. Means and standard deviations for the media use measure by the five twin groups arising from sex and zygosity

*Note:* General= general media use, Victimisation= online victimization, Problematic= problematic media use

**Table S6.** Bivariate twin analyses for media use and mental health phenotypic correlations

*Note:* Victimisation= online victimization, Problematic= problematic media use, General= general media use,; Prosocial= total prosocial behaviour score derived from the subscale of the Strength and Difficulties Questionnaire (SDQ); Depression= Depression scores measured by the mood and feelings questionnaire; Behav= total behavioural problems score derived from the subscale of the SDQ; Anxiety= total anxiety score derived from the subscale of the SDQ ; Conduct= total conduct disorder score derived from the subscale of the SDQ; Peer = total peer problems score derived from the subscale of the SDQ; Prosocial = prosocial behaviour score derived from the subscale of the SDQ; Hyper= total hyperactivity score derived from the subscale of the SDQ; rph= phenotypic correlation; rA= correlation between additive genetic effects; rC= correlation between shared environmental effects; rE= correlation between unique/non-shared environmental effects and measurement error; rphA= proportion on phenotypic correlation accounted for by additive genetic factors; rphC= proportion of phenotypic correlation accounted for by shared environmental factors; rphE= proportion of phenotypic correlation accounted for by unique/non-shared environmental factors and measurement error. Confidence intervals are presented under each estimate in brackets; %rphA = percent of the phenotypic correlation attributed to additive genetic effects; %rphC = percent of the phenotypic correlation attributed to shared environmental effects; %rphE = percent of the phenotypic correlation attributed to unique/non-shared environmental effects and measurement error.

Table S7. Single Nucleotide Polymorphism (SNP) heritability and twin heritability estimates used for genetic sensitivity analyses (Gsens)

| **Phenotype** | **SNP heritability** | **Reference** | **Twin heritability** | **Reference** |
| --- | --- | --- | --- | --- |
| Depression | 0.089(0.003) | Howard, D. M. et al., (2019). *Nature neuroscience*, *22*(3), 343-352. | 0.37 | Sullivan, P. F.et al., (2000). *American journal of psychiatry*, *157*(10), 1552-1562 |
| Anxiety | 0.10 | Cheesman, R. et al., *Translational psychiatry*, *8*(1), 1-9. | 0.26 | Polderman, T. J. Et al., (2015). *Nature genetics*, *47*(7), 702-709. |
| ADHD | 0.22 | Demontis, D. et al.,. (2019). *Nature genetics*, *51*(1), 63-75. | 0.80 | Larsson, H. et al., (2014). *Psychological medicine*, *44*(10), 2223-2229. |

**Supplementary Figures**

**Figure S1a**. Nominal values for the phenotypic correlations between mental health measures and measures of online media use in girls only


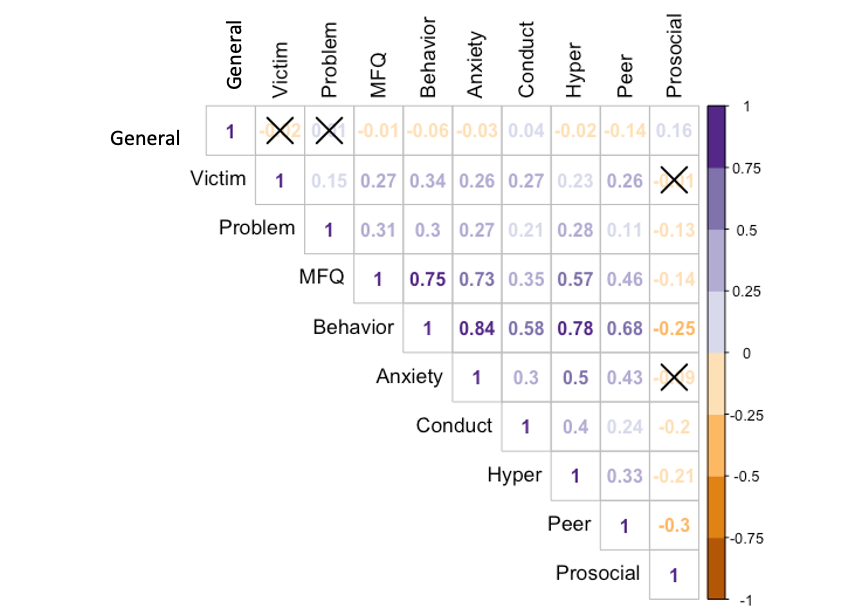


**Figure S1b.** Nominal values for the phenotypic correlations between mental health measures and measures of online media use in boys only


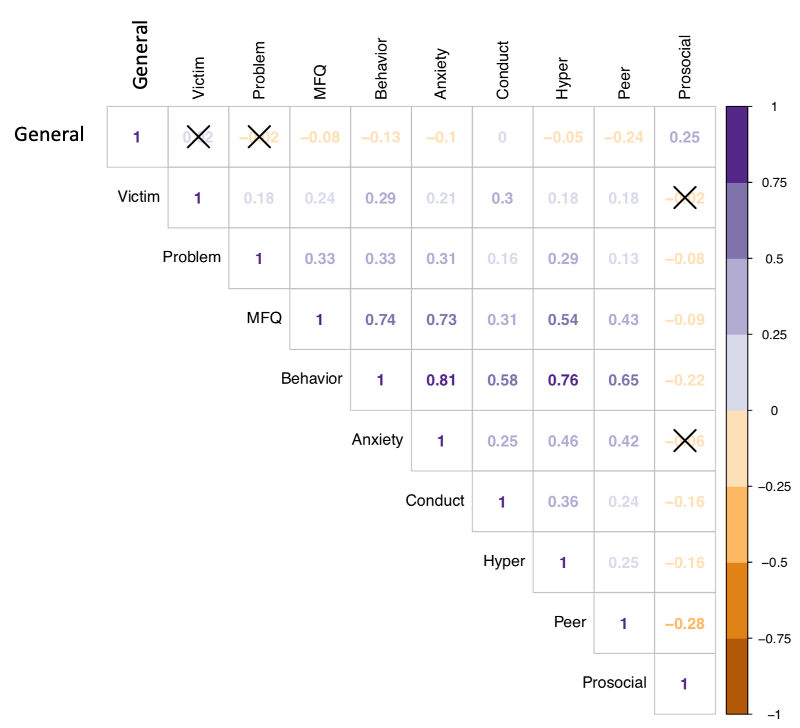


**Figure S2.** Genome-wide polygenic score (GPS) correlations between all 9 GPS and measures of online media use

*Note:* General= general media use; Victim= online victimization; Problem = problematic media use; BPD= bipolar disorder; AN= anorexia; MDD= major depressive disorder; OCD= obsessive compulsive disorder; PTSD= post-traumatic stress disorder; SCHZ= schizophrenia; ADHD= attention deficit hyperactivity disorder; ASD= autism spectrum disorder; EA3= years of education. The size and darkness of the circle represents the strength of the correlation, with blue circles representing higher correlations between traits. The left diagonal gives the nominal value of the correlation. All correlations have been adjusted for multiple testing using the false discovery rate (FDR) method, with correlations that do not survive FDR correction indicated by a blank squar

**Figure S3.** Genetic confounding in the association between problematic media use and depressive symptoms when adjusting for observed polygenic scores for depression


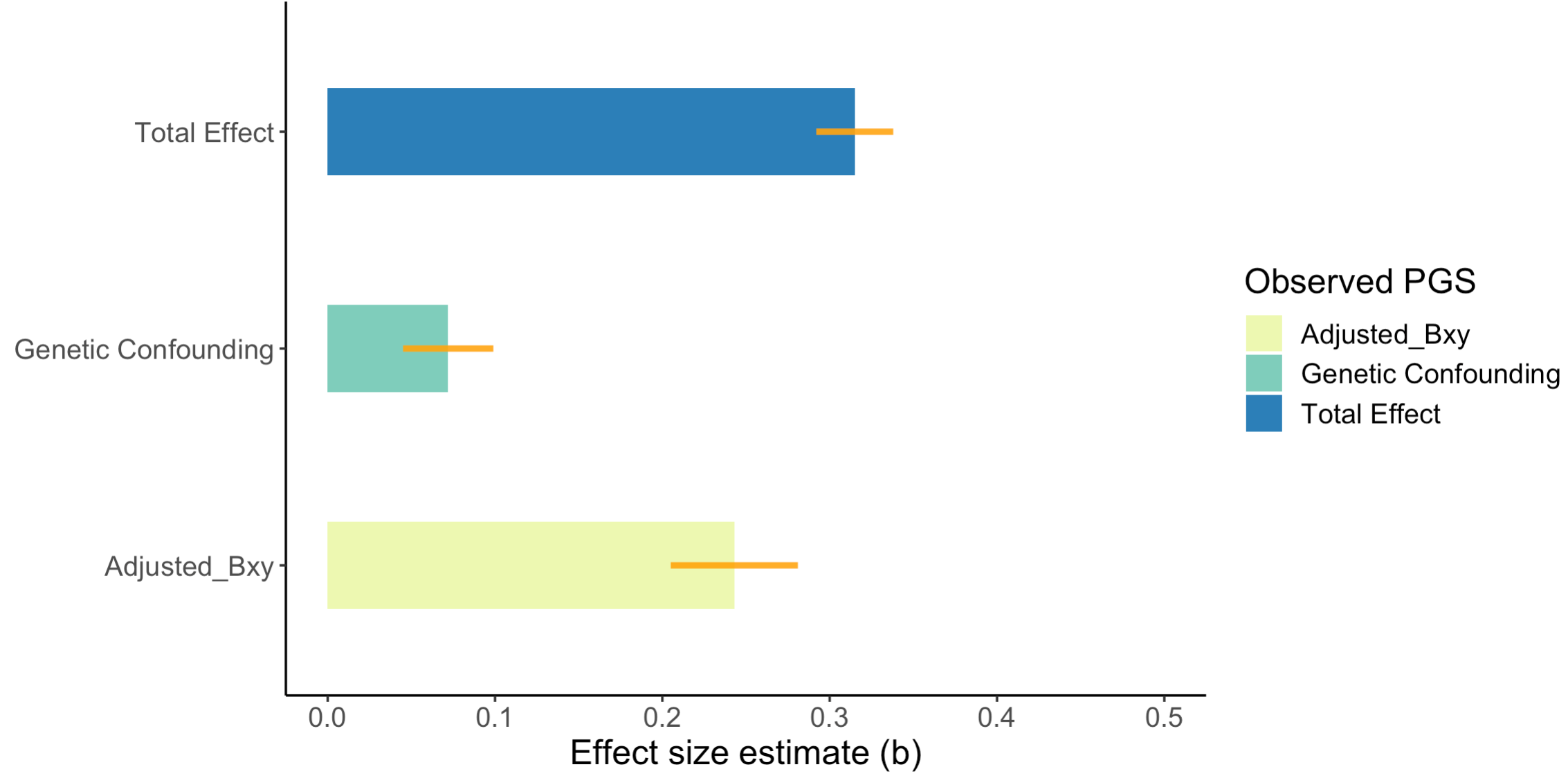


*Note: Adjusted_Bxy =* *standardized estimate of the relationship between problematic media use and depressive symptoms, adjusted for the polygenic score for depression;Genetic Confounding = estimate of genetic confounding; Total Effect = total effect, which adds up to the observed initial association between problemati media use and depressive symptoms.*

**Figure S4.** Genetic confounding in the association between problematic media use and depressive symptoms under a scenario in which the depression polygenic score explains SNP-heritability in depression


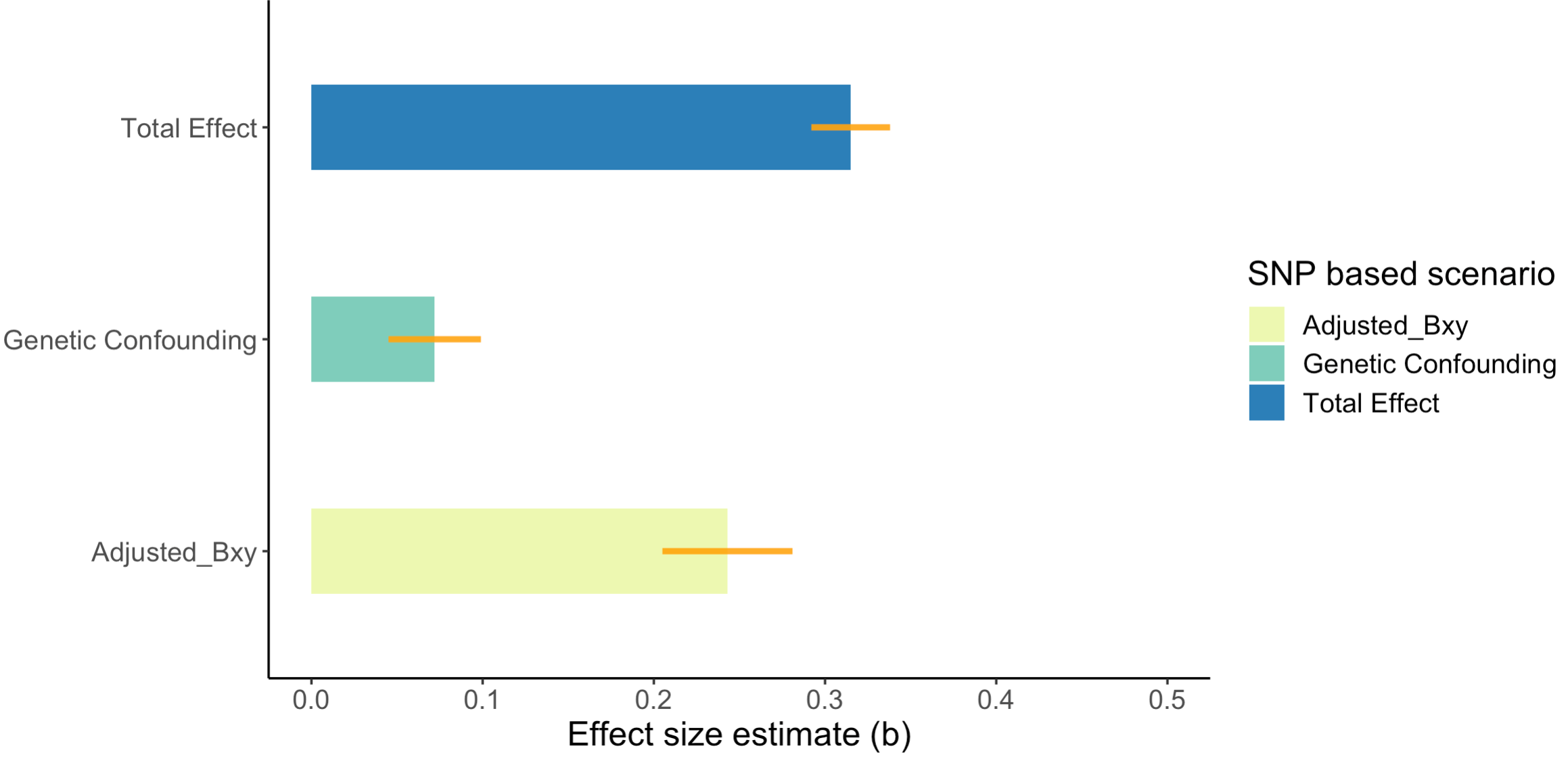


*Note:* Adjusted_Bxy = standardized estimate of the relationship between problematic media use and depressive symptoms, adjusted for a polygenic score that explains SNP-heritability for depression;Genetic Confounding = estimate of genetic confounding; Total Effect = total effect, which adds up to the observed initial association between problematic media use and depressive symptoms.

**Figure S5.** Genetic confounding in the association between problematic media use and depressive symptoms under a scenario in which the depression polygenic score explains twin-heritability in depression


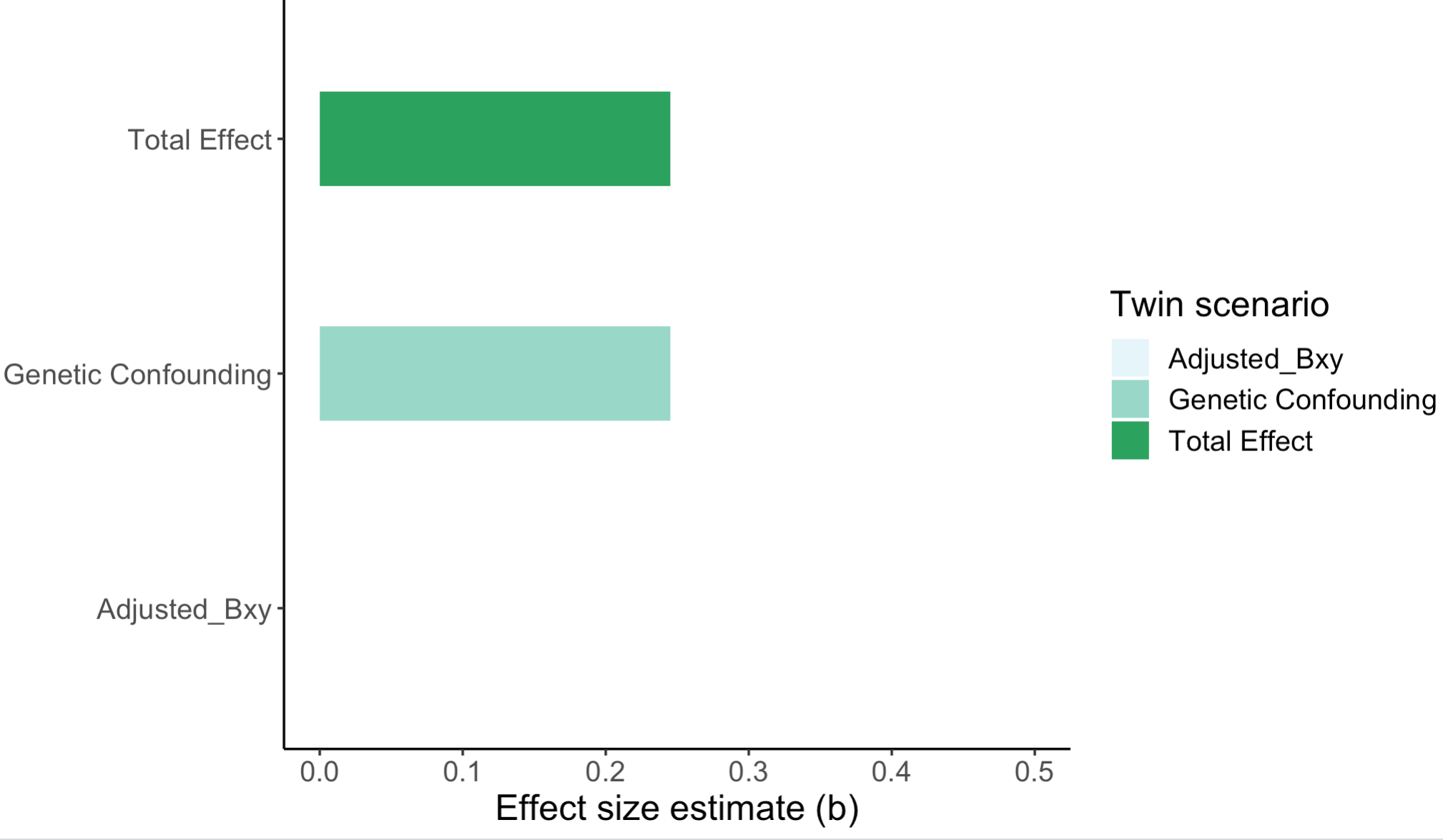


*Note:* Adjusted_Bxy = standardized estimate of the relationship between problematic media use and depressive symptoms, adjusted for a polygenic score that explains twin-heritability for depression;Genetic Confounding = estimate of genetic confounding; Total Effect = total effect, which adds up to the observed initial association between problematic media use and depressive symptoms.

**Figure S6.** Genetic confounding in the association between problematic media use and anxiety when adjusting for observed polygenic scores for anxiety


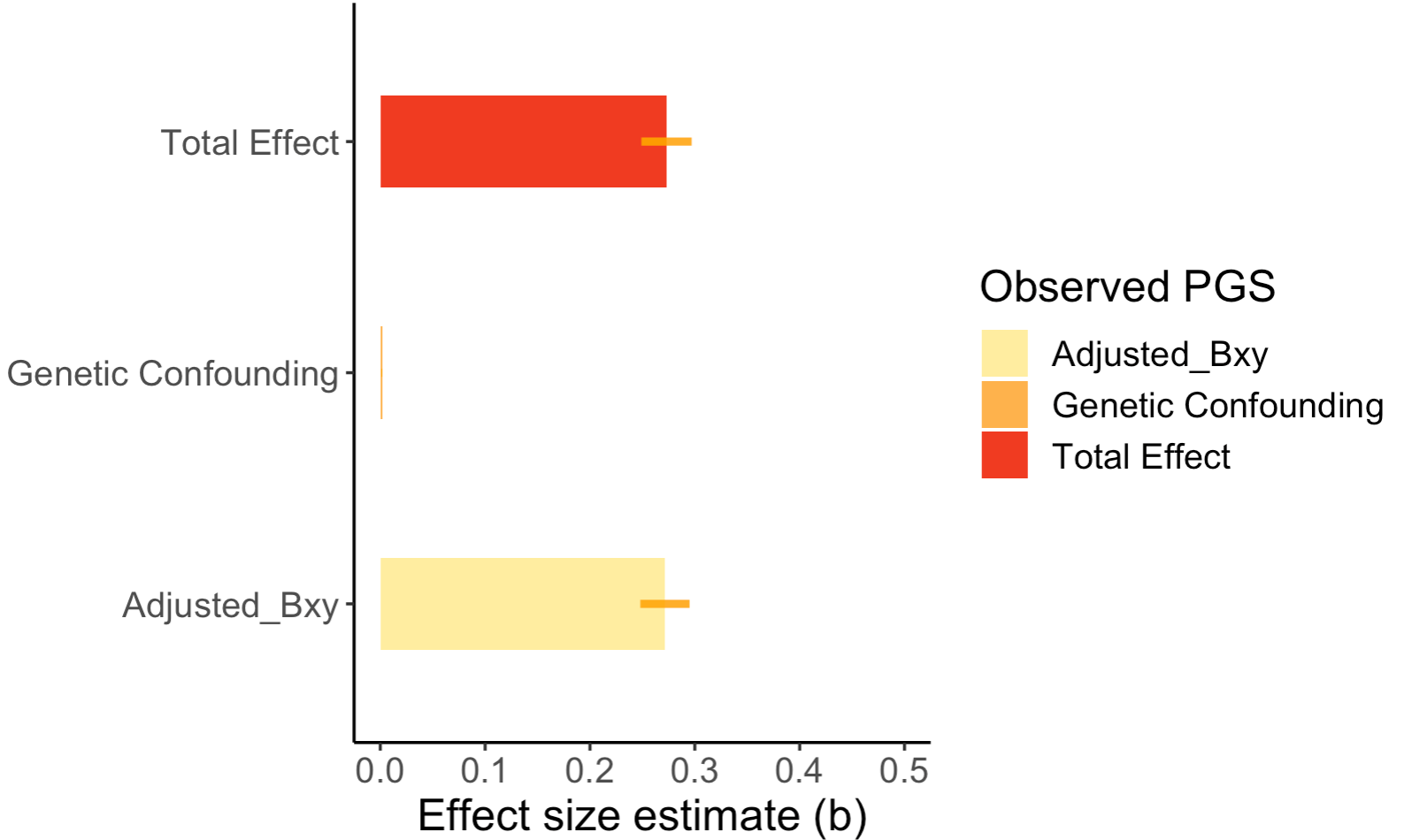


*Note: Adjusted_Bxy =* *standardized estimate of the relationship between problematic media use and anxiety symptoms, adjusted for the polygenic score for anxiety;Genetic Confounding = estimate of genetic confounding; Total Effect = total effect, which adds up to the observed initial association between problematic media use axiety symptoms.*

**Figure S7.** Genetic confounding in the association between problematic media use and anxiety symptoms under a scenario in which the anxiety polygenic score explains SNP-heritability in anxiety


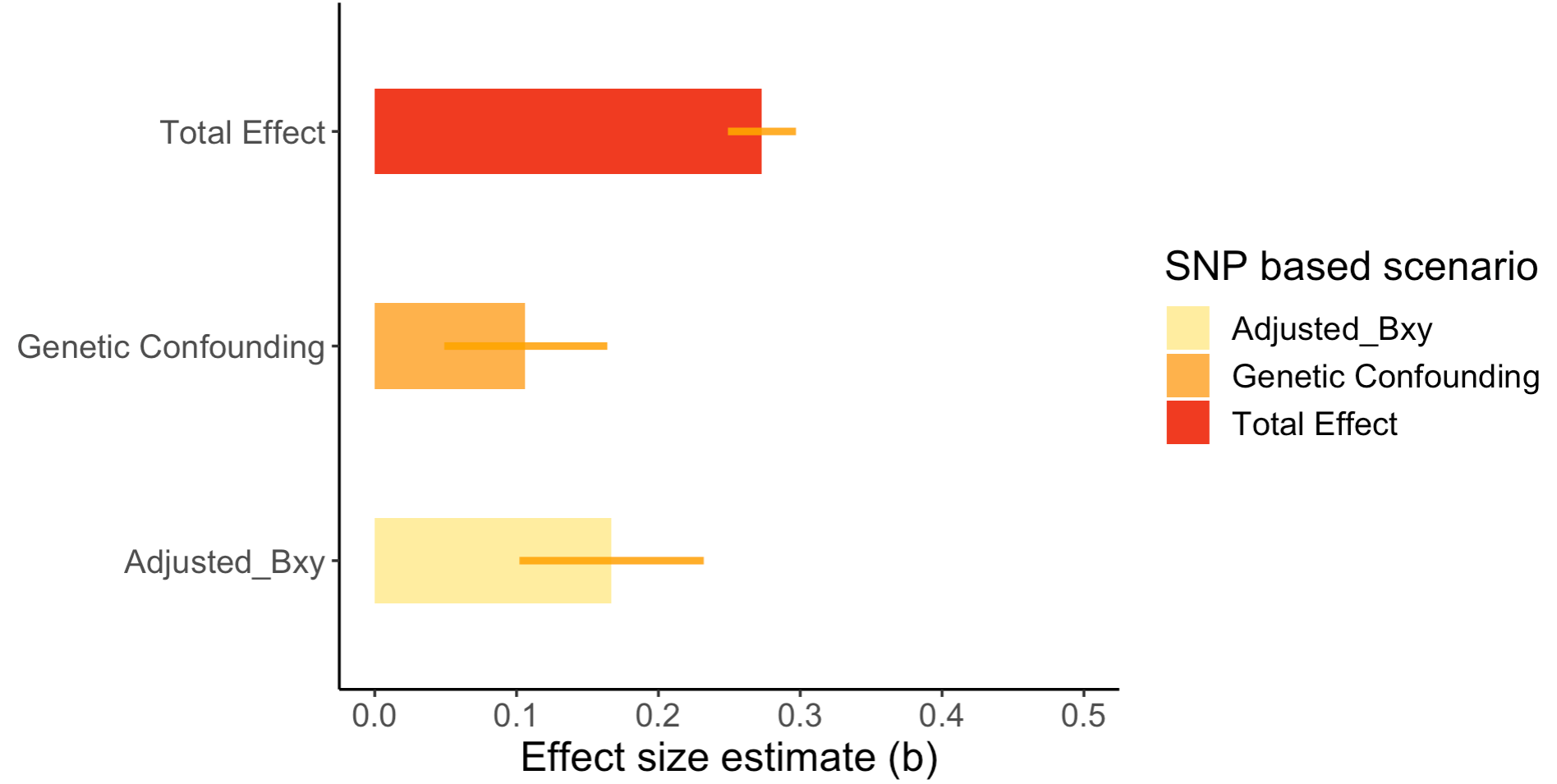


*Note:* Adjusted_Bxy = standardized estimate of the relationship between problematic media use and anxiety symptoms, adjusted for a polygenic score that explains SNP-heritability for anxiety;Genetic Confounding = estimate of genetic confounding; Total Effect = total effect, which adds up to the observed initial association between problematic media use and anxiety symptoms.

**Figure S8.** Genetic confounding in the association between problematic media use and anxiety symptoms under a scenario in which the anxiety polygenic score explains twin-heritability in anxiety


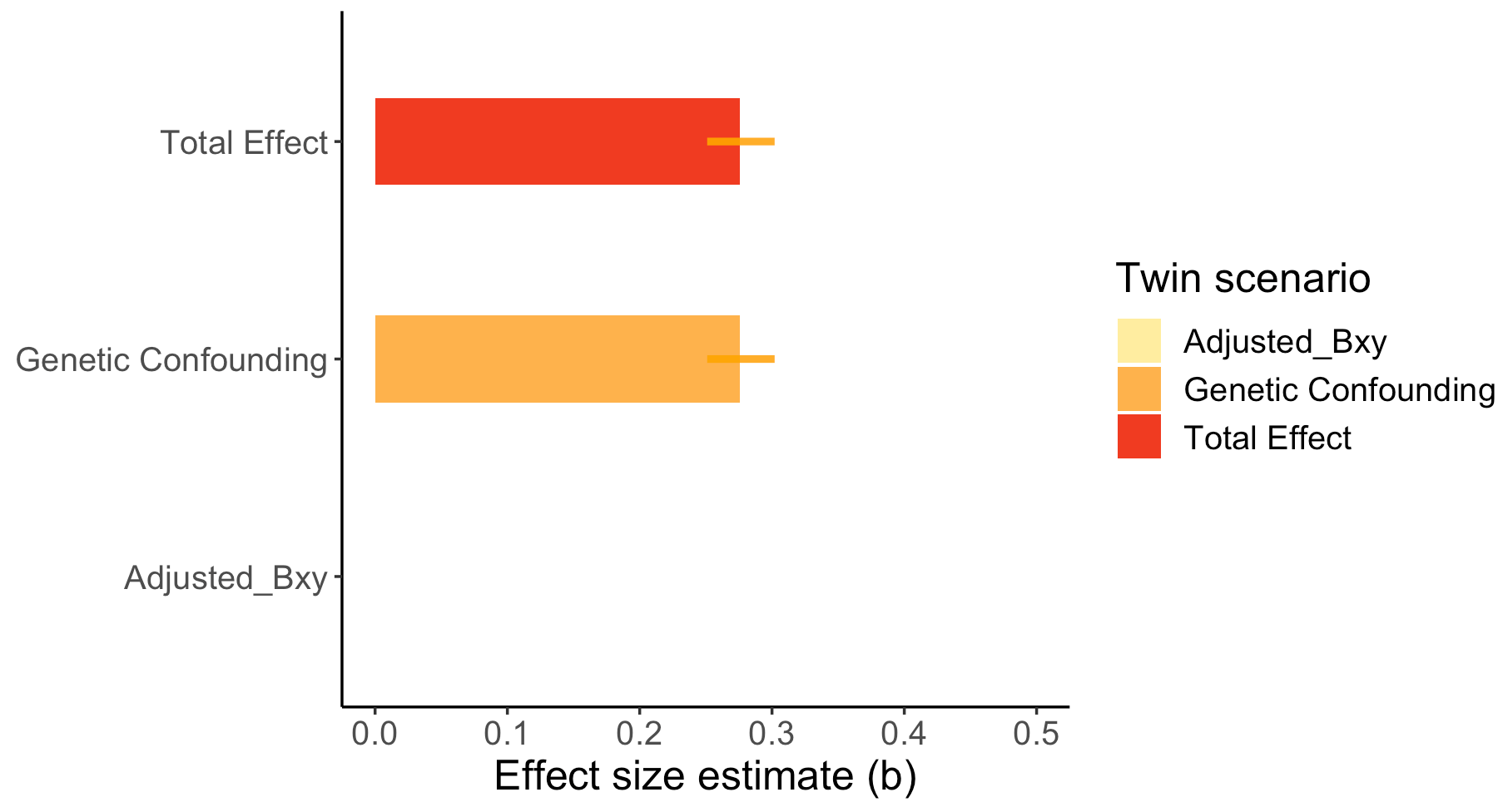


*Note:* Adjusted_Bxy = standardized estimate of the relationship between problematic media use and anxiety symptoms, adjusted for a polygenic score that explains twin-heritability for anxiety;Genetic Confounding = estimate of genetic confounding; Total Effect = total effect, which adds up to the observed initial association between problematic media use and anxiety symptoms.

**Figure S9.** Genetic confounding in the association between online victimisation and anxiety when adjusting for observed polygenic scores for anxiety


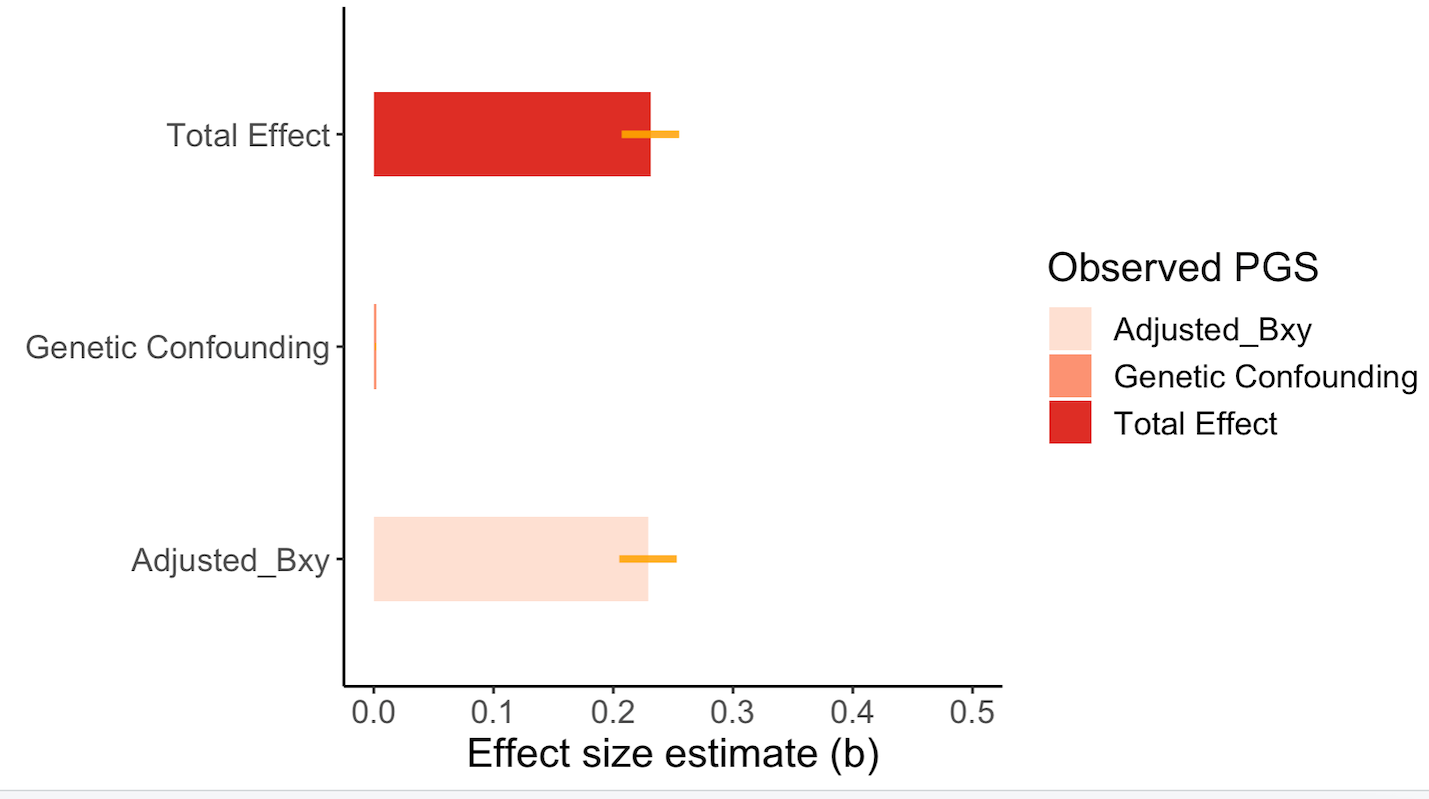


*Note: Adjusted_Bxy =* *standardized estimate of the relationship between online vcitimisation and anxiety symptoms, adjusted for the polygenic score for anxiety;Genetic Confounding = estimate of genetic confounding; Total Effect = total effect, which adds up to the observed initial association between online victimisation and anxiety symptoms.*

**Figure S10.** Genetic confounding in the association between online victimisation and anxiety symptoms under a scenario in which the anxiety polygenic score explains SNP-heritability in anxiety


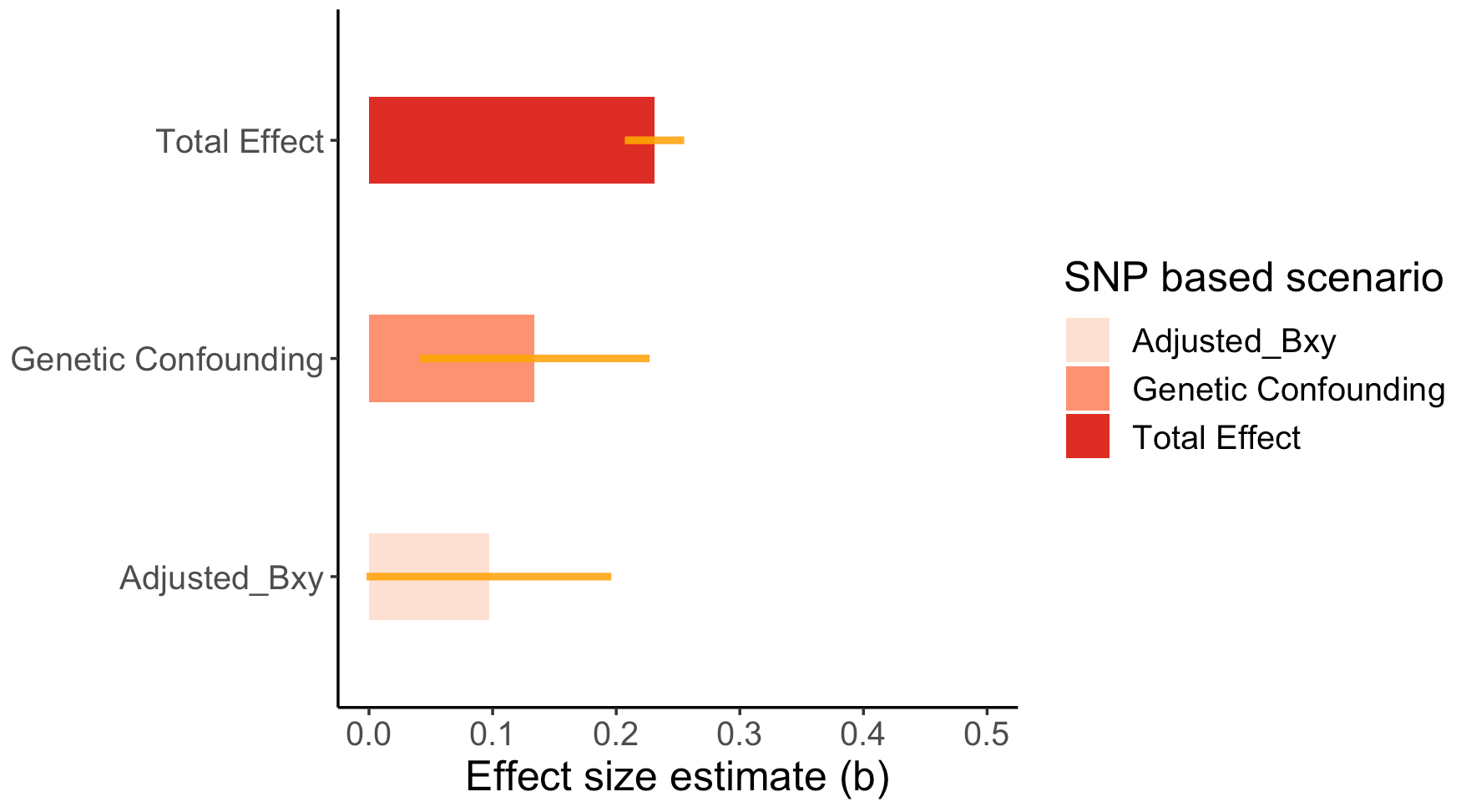


*Note:* Adjusted_Bxy = standardized estimate of the relationship between online victimisation and anxiety symptoms, adjusted for a polygenic score that explains SNP-heritability for anxiety;Genetic Confounding = estimate of genetic confounding; Total Effect = total effect, which adds up to the observed initial association between problematic media use and anxiety symptoms.

**Figure S11.** Genetic confounding in the association between online victimisation and anxiety symptoms under a scenario in which the anxiety polygenic score explains twin-heritability in anxiety


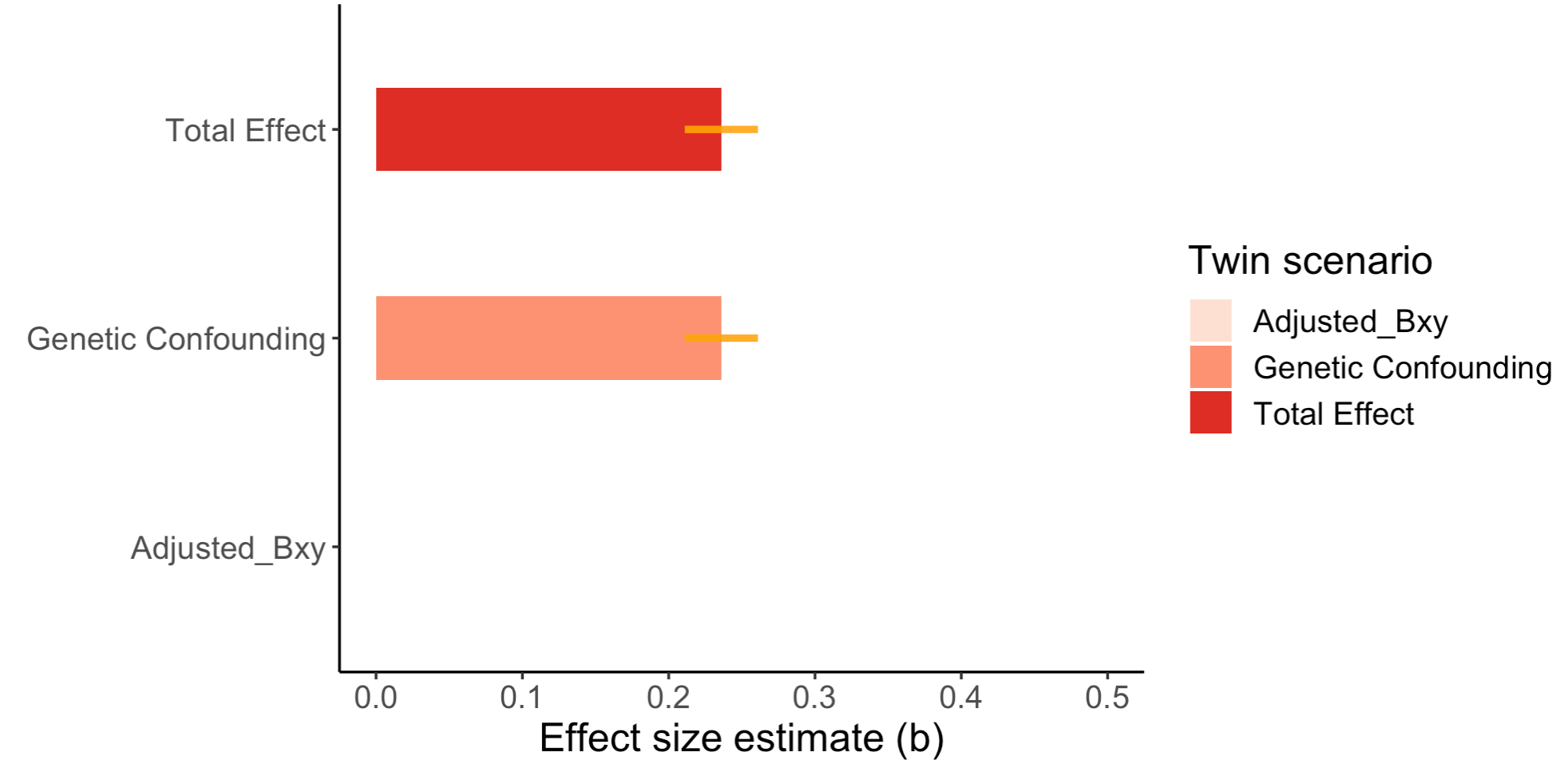


*Note:* Adjusted_Bxy = standardized estimate of the relationship between online victimisation and anxiety symptoms, adjusted for a polygenic score that explains twin-heritability for anxiety;Genetic Confounding = estimate of genetic confounding; Total Effect = total effect, which adds up to the observed initial association between online victimisation and anxiety symptoms.

**Figure S12.** Genetic confounding in the association between problematic media use and hyperactivity when adjusting for observed polygenic scores for hyperactivity


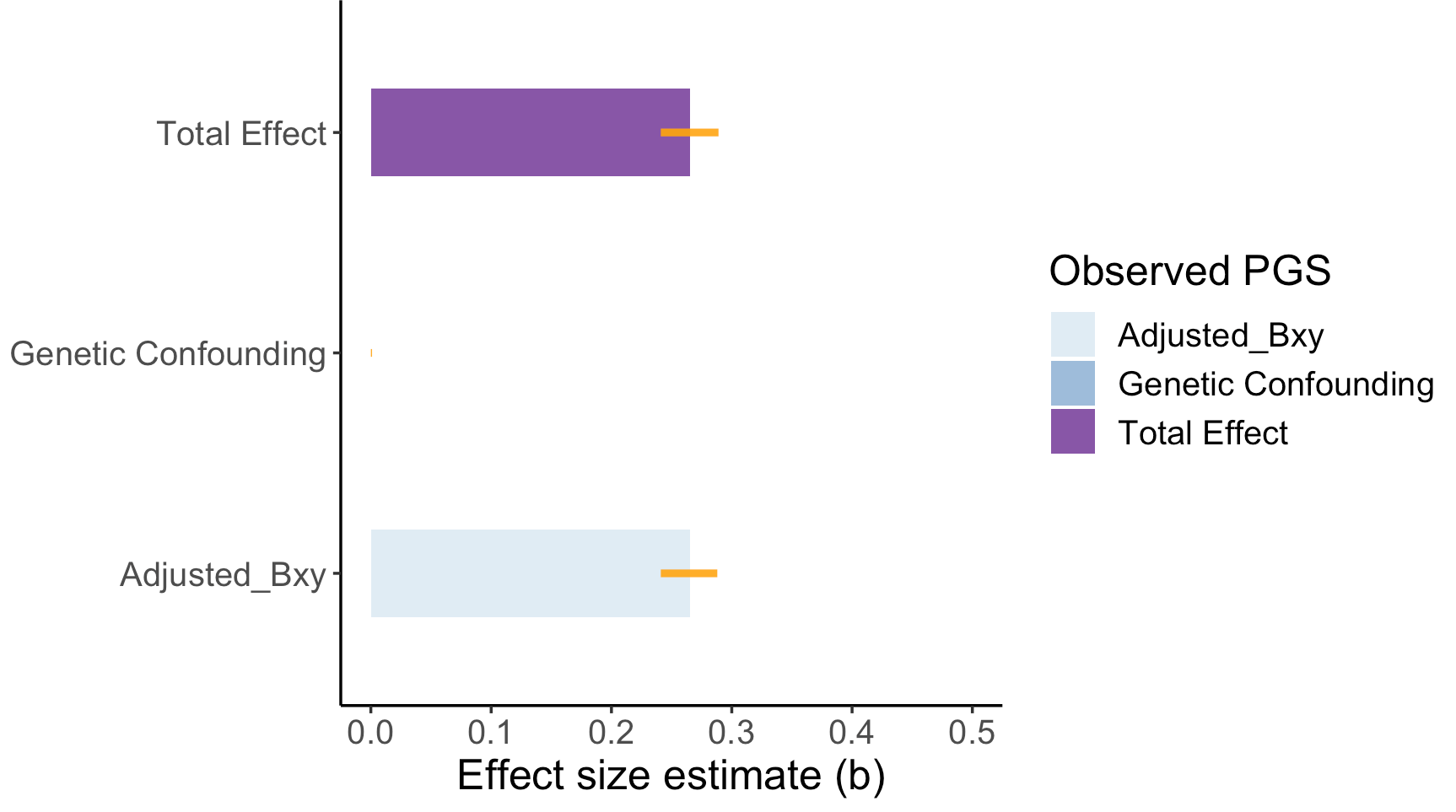


*Note: Adjusted_Bxy =* *standardized estimate of the relationship between problematic media use and hyperactivty symptoms, adjusted for the polygenic score for hyperactivity;Genetic Confounding = estimate of genetic confounding; Total Effect = total effect, which adds up to the observed initial association between problematic media use and hyperactivity symptoms.*

**Figure S13.** Genetic confounding in the association between problematic media use and hyperactivity symptoms under a scenario in which the hyperactivity polygenic score explains SNP-heritability in hyperactivity


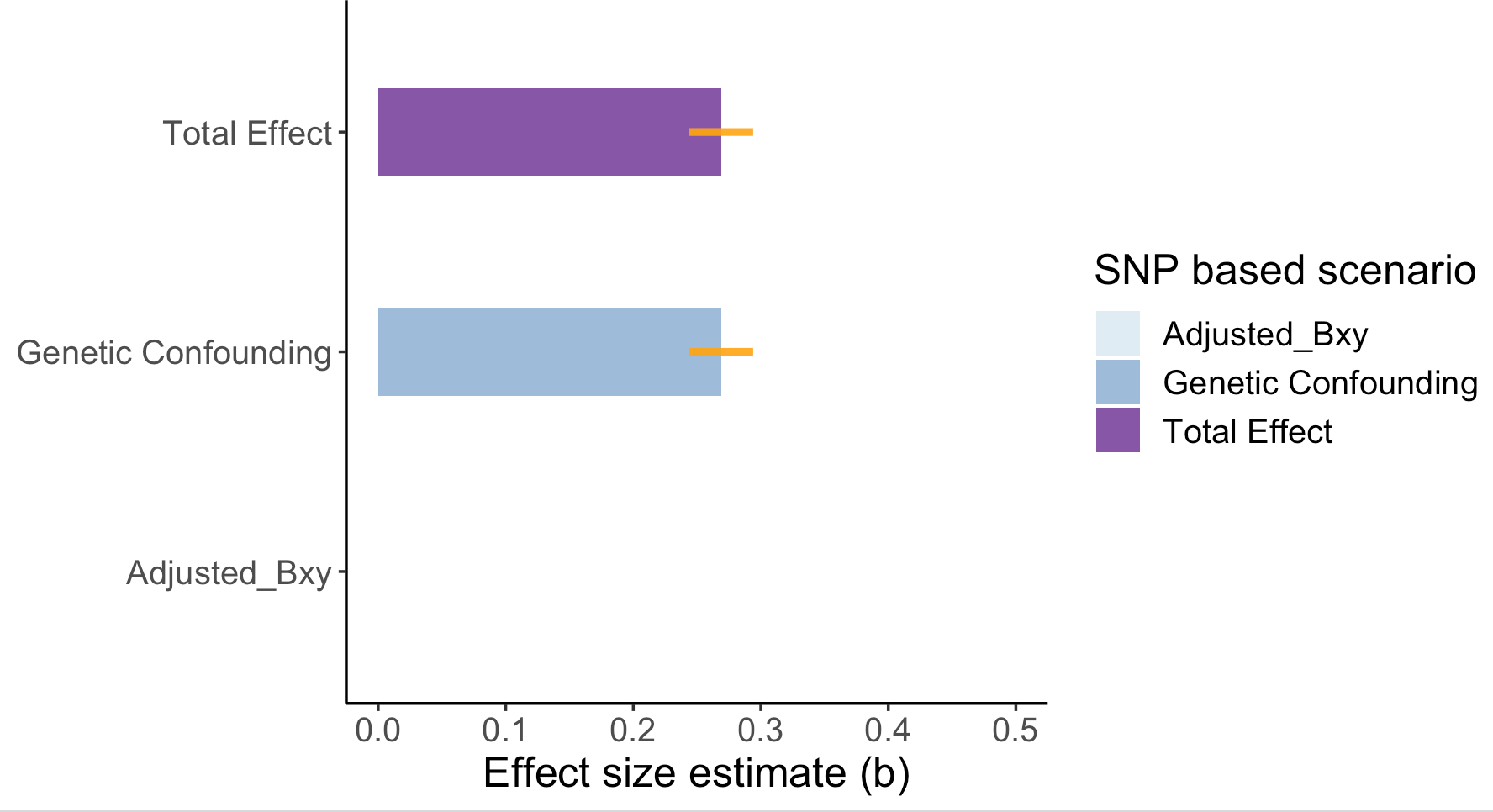


*Note:* Adjusted_Bxy = standardized estimate of the relationship between problematic media use and hyperactivity symptoms, adjusted for a polygenic score that explains SNP-heritability for hyperactivity;Genetic Confounding = estimate of genetic confounding; Total Effect = total effect, which adds up to the observed initial association between problematic media use and hyperactivity symptoms.

**Figure S14.** Genetic confounding in the association between problematic media use and hyperactivity symptoms under a scenario in which the hyperactivity polygenic score explains twin-heritability in hyperactivity


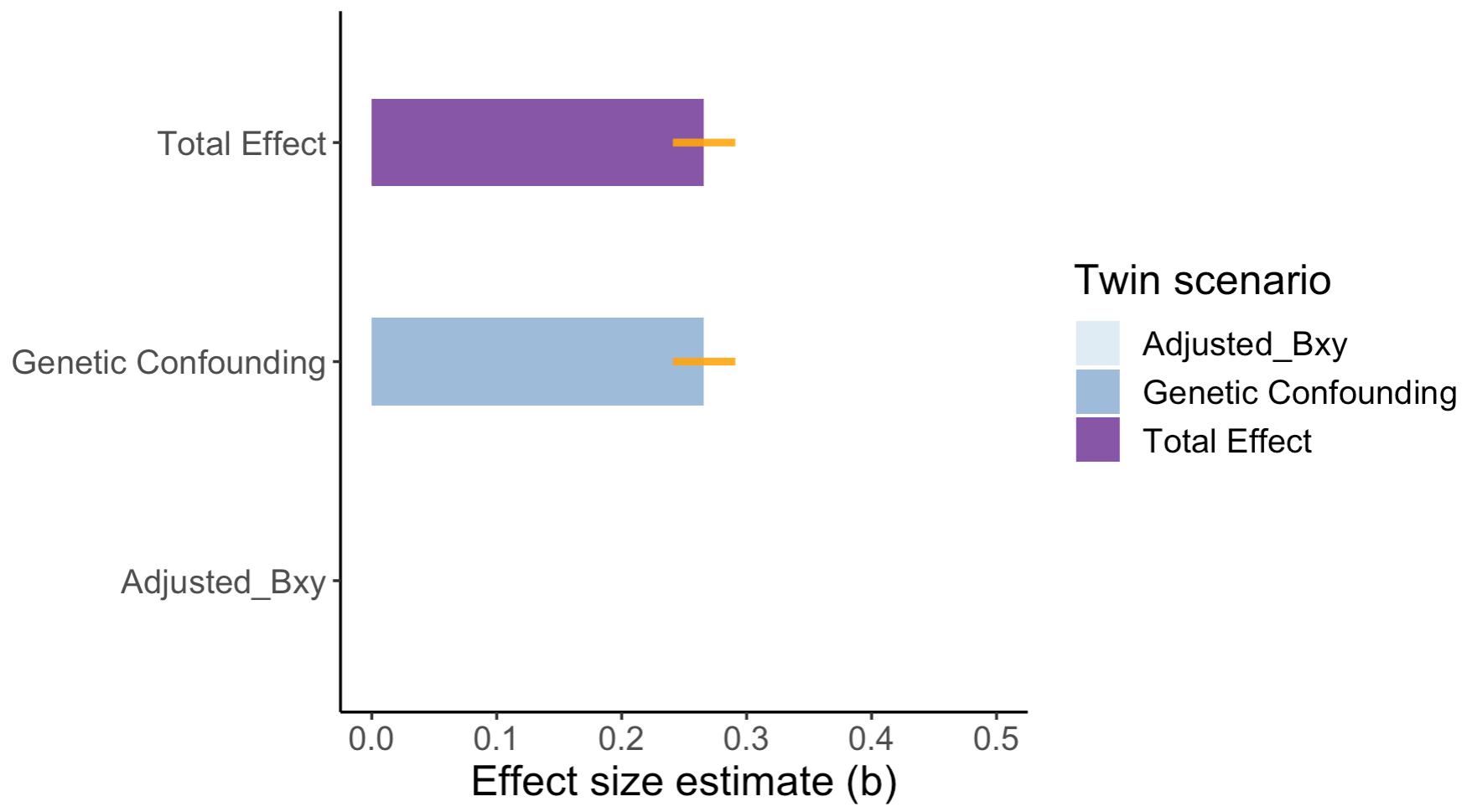


*Note:* Adjusted_Bxy = standardized estimate of the relationship between problematic media use and hyperactivity symptoms, adjusted for a polygenic score that explains twin-heritability for hyperactivity;Genetic Confounding = estimate of genetic confounding; Total Effect = total effect, which adds up to the observed initial association between problematic media use and hyperactivity symptoms.

**Figure S15.** Genetic confounding in the association between online victimisation and hyperactivity when adjusting for observed polygenic scores for hyperactivity


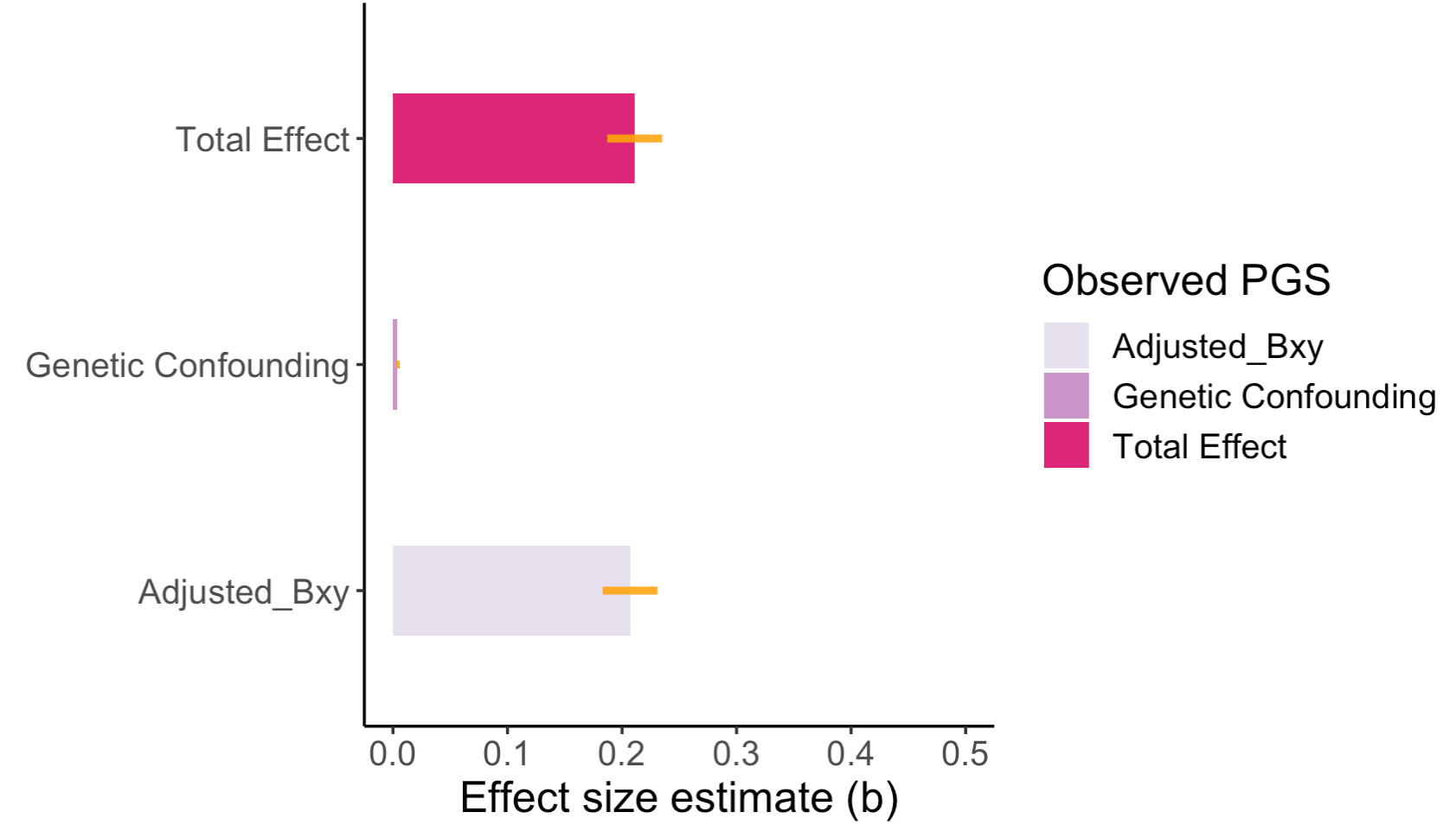


*Note: Adjusted_Bxy =* *standardized estimate of the relationship between online victimisation and hyperactivty symptoms, adjusted for the polygenic score for hyperactivity;Genetic Confounding = estimate of genetic confounding; Total Effect = total effect, which adds up to the observed initial association between online victimisation and hyperactivity symptoms.*

**Figure S16.** Genetic confounding in the association between online victimisation and hyperactivity symptoms under a scenario in which the hyperactivity polygenic score explains SNP-heritability in hyperactivity


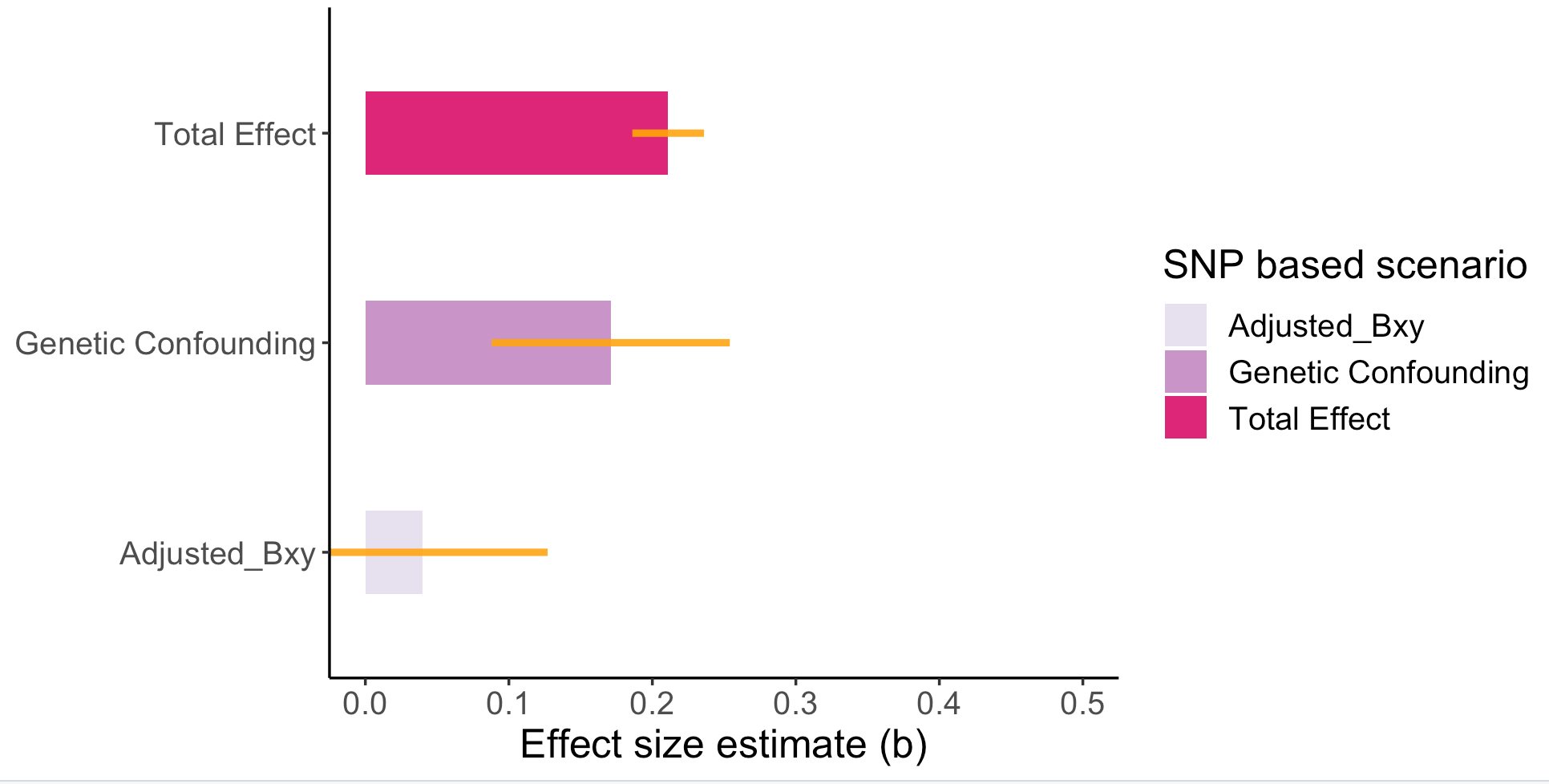


*Note:* Adjusted_Bxy = standardized estimate of the relationship between online victimisation and hyperactivity symptoms, adjusted for a polygenic score that explains SNP-heritability for hyperactivity;Genetic Confounding = estimate of genetic confounding; Total Effect = total effect, which adds up to the observed initial association between online victimisation and hyperactivity symptoms.

**Figure S17.** Genetic confounding in the association between online victimisation and hyperactivity symptoms under a scenario in which the hyperactivity polygenic score explains twin-heritability in hyperactivity


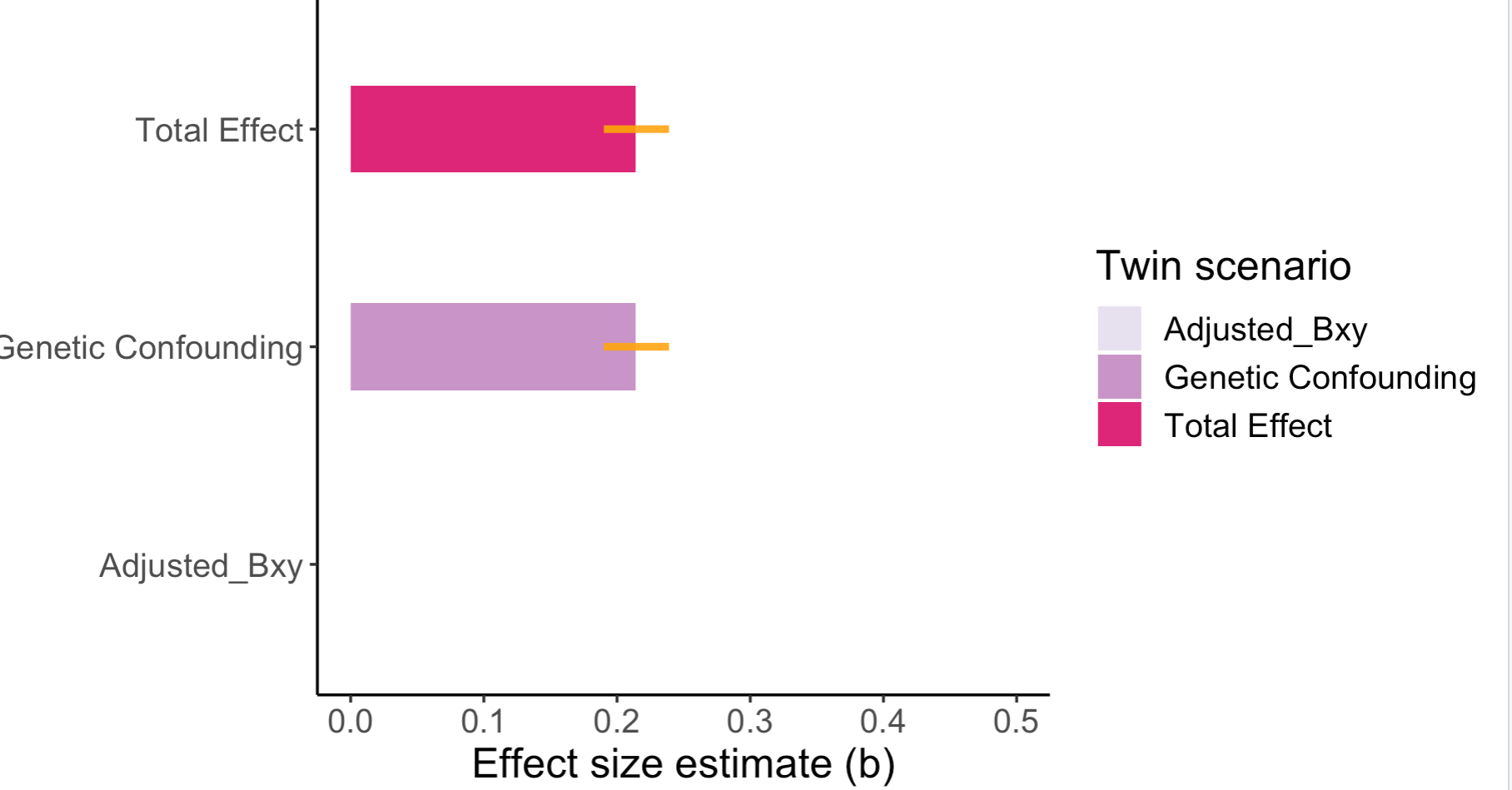


*Note:* Adjusted_Bxy = standardized estimate of the relationship between online victimisation and hyperactivity symptoms, adjusted for a polygenic score that explains twin-heritability for hyperactivity;Genetic Confounding = estimate of genetic confounding; Total Effect = total effect, which adds up to the observed initial association between online victimisation and hyperactivity symptoms.

Figure S18. Full model output for Gsens sensitivity analyses

References

1. Purcell S, Neale B, Todd-Brown K, Thomas L, Ferreira MA, Bender D, et al. PLINK: a tool set for whole-genome association and population-based linkage analyses. Am J Hum Genet. 2007;81(3):559–75.

2. Team RC. R: A language and environment for statistical computing [Internet]. Vienna, Austria: R Foundation for Statistical Computing; 2014. 2015;

3. Li H. A statistical framework for SNP calling, mutation discovery, association mapping and population genetical parameter estimation from sequencing data. Bioinformatics. 2011;27(21):2987–93.

4. Patterson N, Price AL, Reich D. Population structure and eigenanalysis. PLoS Genet. 2006;2(12):e190.

5. Price AL, Patterson NJ, Plenge RM, Weinblatt ME, Shadick NA, Reich D. Principal components analysis corrects for stratification in genome-wide association studies. Nat Genet. 2006;38(8):904.

6. Loh P-R, Danecek P, Palamara PF, Fuchsberger C, Reshef YA, Finucane HK, et al. Reference-based phasing using the Haplotype Reference Consortium panel. Nat Genet. 2016;48(11):1443.

7. McCarthy S, Das S, Kretzschmar W, Durbin R, Abecasis G, Marchini J. A reference panel of 64,976 haplotypes for genotype imputation. bioRxiv. 2016;

8. Durbin R. Efficient haplotype matching and storage using the positional Burrows–Wheeler transform (PBWT). Bioinformatics. 2014;30(9):1266–72.

9. Fuchsberger C, Abecasis GR, Hinds DA. minimac2: faster genotype imputation. Bioinformatics. 2015;31(5):782–4.

10. Vilhjálmsson BJ, Yang J, Finucane HK, Gusev A, Lindström S, Ripke S, et al. Modeling linkage disequilibrium increases accuracy of polygenic risk scores. Am J Hum Genet. 2015;97(4):576–92.

11. Vattikuti S, Guo J, Chow CC. Heritability and genetic correlations explained by common SNPs for metabolic syndrome traits. PLoS Genet. 2012;8(3):e1002637.

12. Benjamini Y, Hochberg Y. Controlling the false discovery rate: a practical and powerful approach to multiple testing. J R Stat Soc Ser B. 1995;57(1):289–300.

13. Lee JJ, Wedow R, Okbay A, Kong E, Maghzian O, Zacher M, et al. Gene discovery and polygenic prediction from a genome-wide association study of educational attainment in 1.1 million individuals. Nat Genet. 2018;1112–1121.
